# Non-lysine ubiquitylation: Doing things differently

**DOI:** 10.3389/fmolb.2022.1008175

**Published:** 2022-09-19

**Authors:** Ian R. Kelsall

**Affiliations:** MRC Protein Phosphorylation and Ubiquitylation Unit, University of Dundee, Scotland, United Kingdom

**Keywords:** ubiquitin, oxyester bond, thioester bond, non-lysine ubiquitylation, ERAD, LUBAC, non-canonical ubiquitylation, Rnf213

## Abstract

The post-translational modification of proteins with ubiquitin plays a central role in nearly all aspects of eukaryotic biology. Historically, studies have focused on the conjugation of ubiquitin to lysine residues in substrates, but it is now clear that ubiquitylation can also occur on cysteine, serine, and threonine residues, as well as on the N-terminal amino group of proteins. Paradigm-shifting reports of non-proteinaceous substrates have further extended the reach of ubiquitylation beyond the proteome to include intracellular lipids and sugars. Additionally, results from bacteria have revealed novel ways to ubiquitylate (and deubiquitylate) substrates without the need for any of the enzymatic components of the canonical ubiquitylation cascade. Focusing mainly upon recent findings, this review aims to outline the current understanding of non-lysine ubiquitylation and speculate upon the molecular mechanisms and physiological importance of this non-canonical modification.

## Introduction

**FIGURE 1:**
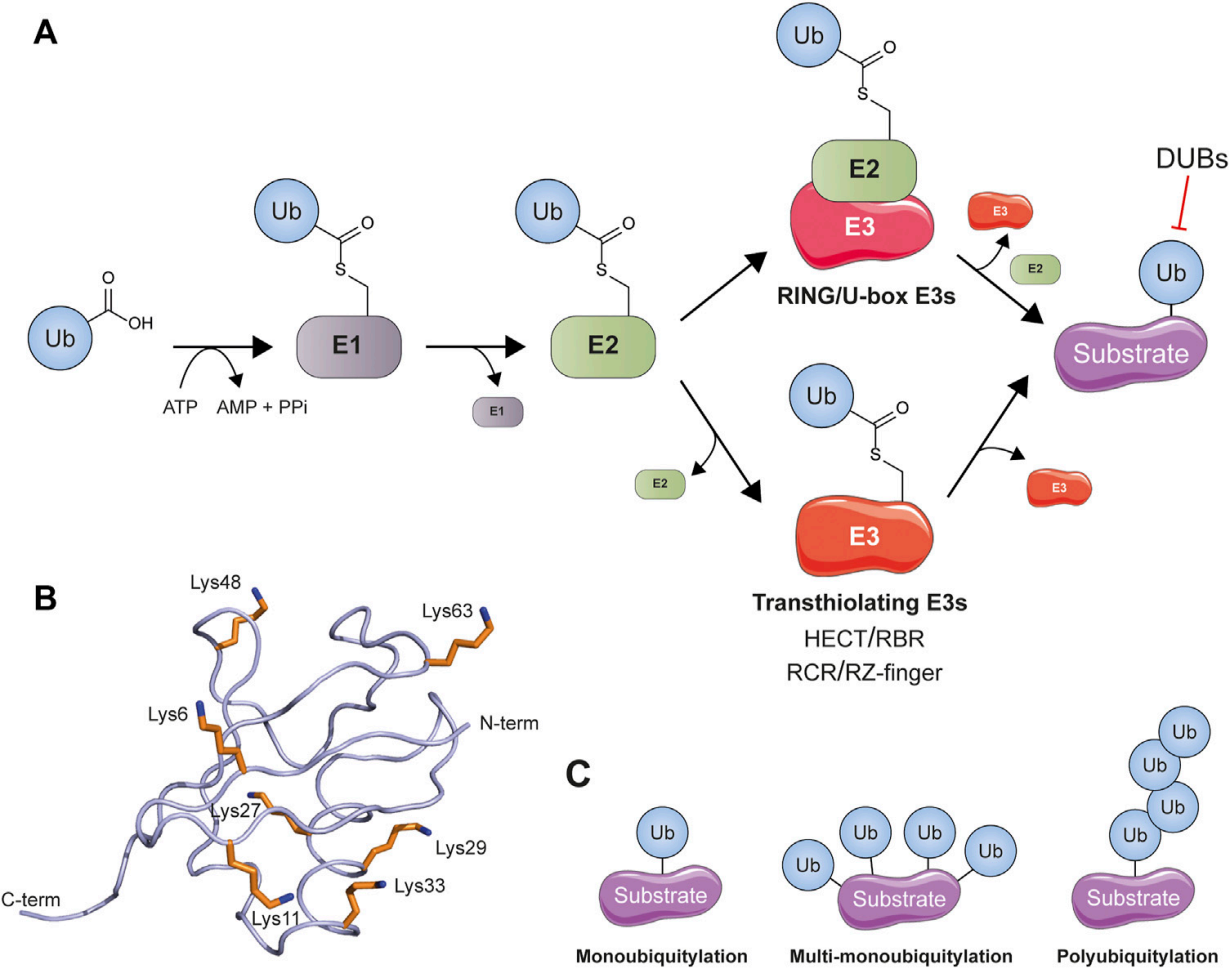
Components of the conventional ubiquitylation cascade. **(A)** A multicomponent enzyme cascade is used to transfer ubiquitin (Ub) to its substrates. RING and U-box ligases facilitate the transfer of ubiquitin from E2 directly to substrate lysine whereas the transthiolating classes of ligase (HECT/RBR/RCR/RZ-finger) first covalently bind ubiquitin at their own active site cysteine before transferring it to substrate. Ubiquitin can be removed by specialized deubiquitylating enzymes (DUBs). **(B)** The structure of ubiquitin (PDB: 1UBQ) (Vijay-Kumar et al., 1987) is shown as a smoothed backbone trace. The position of ubiquitin’s seven lysine residues is indicated. **(C)** Different ubiquitylation topologies are possible: monoubiquitylation, multi-monoubiquitylation, and polyubiquitylation (in which ubiquitin can ubiquitylate itself to form amide-linked chains) all occur.

The post-translational modification of proteins is a method used throughout nature to dramatically expand the complexity and plasticity of the encoded proteome (Walsh et al., 2005). Ubiquitylation, the covalent attachment of the small globular protein ubiquitin to substrate proteins, is an example of one such versatile and highly complex modification. The process of ubiquitylation is achieved through the orchestrated action of three distinct classes of enzyme (Figure 1A). In an ATP-dependent first step the C-terminal carboxy group of ubiquitin is attached to a catalytic cysteine residue in a ubiquitin-activating (E1) enzyme (Ciechanover et al., 1981; Hershko et al., 1981). This ‘activated’ ubiquitin is then transferred onto the active-site cysteine of a ubiquitin-conjugating (E2) enzyme via a transthiolation reaction (Hershko et al., 1983). Finally, the E2 cooperates with a ubiquitin ligase (E3) enzyme to facilitate the transfer of ubiquitin to the substrate, canonically by the formation of an amide (isopeptide) bond between the C-terminal carboxy group of ubiquitin and the ε-amino group of a lysine residue in the target protein (Pickart 2001). With over 600 members encoded in the human genome (Li et al., 2008) E3 ligases represent the most numerous enzyme family in the ubiquitylation cascade, but they may be further divided into several smaller subfamilies based upon protein fold and mechanism of action (Berndsen and Wolberger 2014). The RING (Really Interesting New Gene) and U-box ligases stimulate direct transfer of ubiquitin to the substrate (Deshaies and Joazeiro 2009) whereas the HECT (Homologous to E6AP Carboxyl Terminus) and RBR (RING-between-RING) ligase families contain a catalytic cysteine residue that undergoes transthiolation to form a thioester-linked intermediate with ubiquitin prior to ubiquitylation of the substrate (Wenzel et al., 2011; Lorenz 2018). Additionally, two new classes of transthiolating E3 have recently been discovered. MYCBP2 (Myc-binding protein 2) represents the first example of an RCR (RING-Cys-Relay) ligase that contains two catalytic cysteine residues that relay ubiquitin to the substrate via thioester intermediates (Pao et al., 2018) while RNF213 (RING Finger Protein 213) utilizes a non-canonical zinc-binding RZ (RNF213-ZNFX1 finger) domain to conjugate ubiquitin via an active-site cysteine residue (Ahel et al., 2021; Otten et al., 2021). Substrate ubiquitylation can take several different forms, sometimes referred to as the ‘ubiquitin code’ (Komander and Rape 2012). Some substrate proteins are ubiquitylated at a single lysine with only a single ubiquitin (monoubiquitylation) while for others this process may be repeated on multiple lysines to give rise to multi-monoubiquitylation. Additional complexity comes from ubiquitin’s ability to self-conjugate to the ε-amines of any of its seven internal lysines, allowing the formation of polyubiquitin chains (Komander 2009) (Figures 1B,C). These conformationally-distinct ubiquitin linkages provide an information-rich architectural scaffold that may be recognized by distinct ubiquitin binding domains to produce diverse biological outcomes (Dikic et al., 2009).

While lysine ubiquitylation may still be considered canonical, atypical non-lysine ubiquitylation is establishing itself as yet another way that this compact 76-amino-acid protein can surprise us with its versatility. It is now clear that ubiquitin can modify cystine, serine and threonine residues, as well as the N-terminal amino group of proteins. Furthermore, recent reports of non-proteinaceous substrates extend the reach of ubiquitylation beyond the proteome, while exciting results from bacteria remove the need for the canonical ubiquitylation machinery entirely, revealing novel ways to couple ubiquitin to substrates. The history of non-lysine ubiquitylation has been charted by a series of comprehensive review articles (Wang X. et al., 2012; McDowell and Philpott 2013; McDowell and Philpott 2016; McClellan et al., 2019; Squair and Virdee 2022) but in view of exciting recent developments it is timely to revisit and update some examples of non-canonical ubiquitylation to discuss emerging themes and consider the future of this burgeoning field.

## Beyond the ubiquitylation of lysine

**FIGURE 2:**
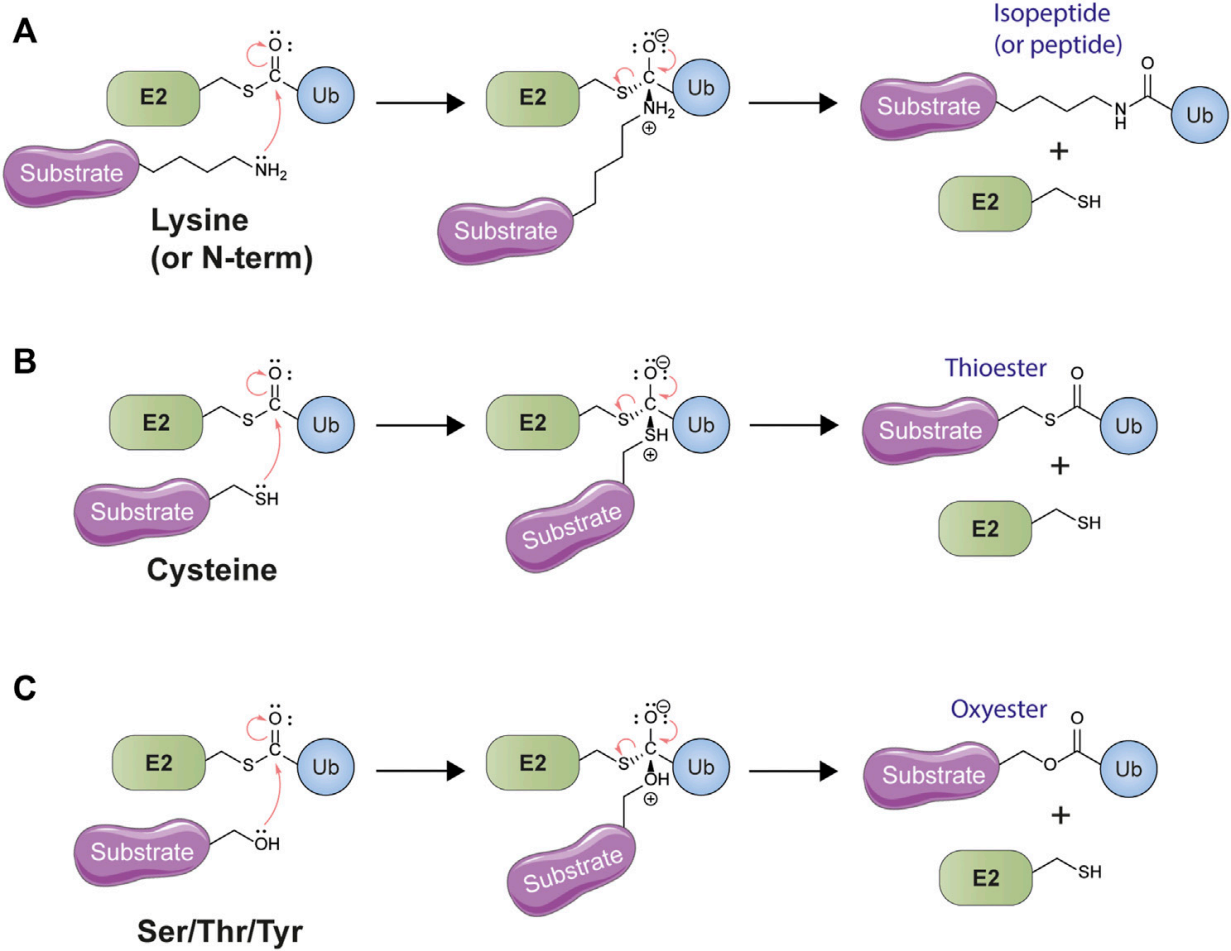
Canonical and non-canonical ubiquitylation both represent examples of nucleophilic acyl transfer. **(A)** Conventional ubiquitylation involves the attack of the electrophilic thioester carbonyl of an E2-ubiquitin (Ub) conjugate by the lone pair electrons of the substrate lysine amine group. This leads to the formation and then collapse of a tetrahedral intermediate, expelling the E2 leaving group and resulting in lysine ubiquitylation. The same mechanism may also be employed to ubiquitylate the free amine group of the N-terminus of the polypeptide backbone. **(B)** Like nitrogen, the cysteine thiol also possesses a lone pair of electrons and can act as a nucleophile in an acyl transfer reaction. This same mechanism is employed by E2s themselves when they accept ubiquitin from the E1 enzyme, as well as by the transthiolating E3 ligases. This reaction leads to the formation of a thioester. **(C)** The amino acids serine, threonine, and tyrosine possess a nucleophilic hydroxyl group which can be ubiquitylated by means of a nucleophilic acyl transfer mechanism. The resulting linkage between ubiquitin and the substrate is an oxyester.

By the mid-to-late 1980s it had become generally accepted within the ubiquitin community that ubiquitylation involved the formation of an isopeptide bond between the C-terminus of ubiquitin and the ε-amino group of a lysine residue in the substrate protein (Busch 1984; Hershko 1988; Chau et al., 1989). Despite this, puzzling examples existed—and would continue to arise—of protein ubiquitylation in the absence of available lysines (Hershko et al., 1984; Hershko and Heller 1985; Hodgins et al., 1996; Brandimarti and Roizman 1997; Yu et al., 1997). In addition, it was recognized early on that the ubiquitylation machinery was highly promiscuous and capable of working with non-native substrates (Rose and Warms 1983; Pickart and Rose 1985a; Pickart and Rose 1985b), a property that has since been exploited in numerous structural and mechanistic studies (see, for example, Sung et al., 1991; Burchak et al., 2006; Eddins et al., 2006; Wenzel et al., 2011; Plechanovova et al., 2012). Isopeptide bond formation during ubiquitylation is an example of an acyl transfer reaction, where the electron-deficient carbonyl carbon of the thioester linkage undergoes nucleophilic attack by the lone pair of electrons on the ε-amino nitrogen atom of the substrate lysine (Figure 2A). In principle this mechanism can also operate with the N-terminal amino group of proteins, as well as with the nucleophilic amino acid side chains of cysteine, serine, threonine, and tyrosine (Figures 2B,C). These latter chemical linkages (thioester and oxyester bonds), though conceptually possible, were initially considered too labile to be of physiological relevance but a substantial body of work accumulated over the past two decades has challenged this assertion.

## N-terminal ubiquitylation

### Met1-linked polyubiquitin

**FIGURE 3:**
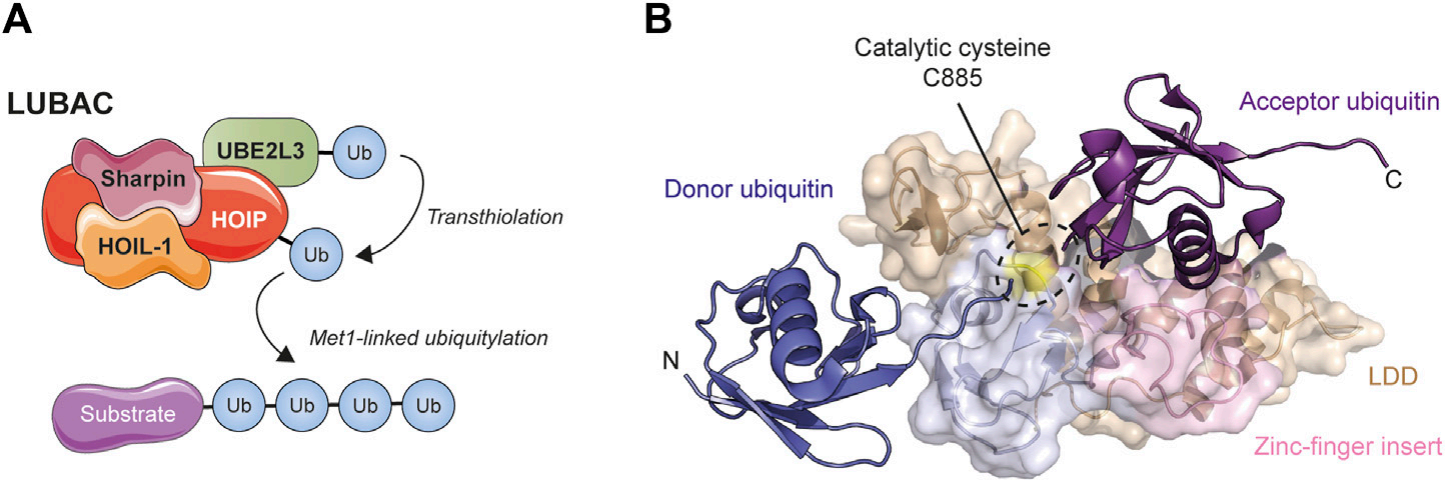
Met1-linked ubiquitin is synthesized by the Linear Ubiquitin Chain Assembly Complex (LUBAC). **(A)** LUBAC is a trimeric complex composed of HOIP, HOIL-1, and Sharpin. HOIP binds its cognate E2 (very likely UBE2L3 (Lewis et al., 2015)) and by means of a transthiolation mechanism that sees ubiquitin transferred to Cys885 in the catalytic RBR domain of the ligase, builds Met1-linked (‘linear’) ubiquitin chains in which peptide bonds between the C- and N-termini of ubiquitin create a single continuous polypeptide chain of repeating ubiquitin units. **(B)** Structure of the minimal catalytic core of HOIP in complex with acceptor (purple) and donor (dark blue) ubiquitin (PDB: 4LJO) (Stieglitz et al., 2013). The unique LDD (wheat colour) and zinc-finger (pink) domains of HOIP help ensure that the C-terminus of the donor ubiquitin and N-terminus of the acceptor ubiquitin align for Met1 linkage formation. Note the proximity of the catalytic cysteine (coloured yellow) to the N-terminus of the acceptor ubiquitin, explaining the specificity for linear chains.

The best characterized example of non-lysine ubiquitylation involves the formation of a peptide bond between the carboxy-terminus of an incoming ‘donor’ ubiquitin and the amino-terminus of the preceding ‘acceptor’ ubiquitin to generate so-called ‘linear’ or Met1-linked polyubiquitin chains, key players in immune signaling and cell death regulation (linear ubiquitylation has been extensively reviewed elsewhere and the reader is directed towards one of the many excellent reviews on the subject, such as Elliott 2016, Hrdinka and Gyrd-Hansen 2017, Rittinger and Ikeda 2017, Dittmar and Winklhofer 2019, Oikawa et al., 2020, Fuseya and Iwai 2021, or Jahan et al., 2021). Met1-linked ubiquitin is specifically generated by the linear ubiquitin chain assembly complex (LUBAC) (Kirisako et al., 2006), the only E3 ligase capable of producing Met1-linked ubiquitin chains *de novo* (Emmerich et al., 2013; Peltzer et al., 2014; Emmerich et al., 2016) (Figure 3A). LUBAC is composed of three proteins—HOIL-1 (Haem-oxidised IRP2 ubiquitin ligase-1), HOIP (HOIL-1-interacting protein), and Sharpin (SHANK-associated RH domain-interacting protein)—and although both HOIL-1 and HOIP possess E3 ubiquitin ligase activity, it is HOIP that represents the enzyme directly responsible for Met1-linked ubiquitin synthesis (Kirisako et al., 2006; Gerlach et al., 2011; Ikeda et al., 2011; Tokunaga et al., 2011; Smit et al., 2012; Stieglitz et al., 2012). HOIP is a member of the transthiolating RBR class of E3 ubiquitin ligases, and as such it is HOIP, and not its cognate E2, that determines ubiquitin chain linkage type (Kirisako et al., 2006; Berndsen and Wolberger 2014). A unique domain known as the linear ubiquitin chain-determining domain (LDD) is located C-terminal to the active-site cysteine, where it orientates the proximal ‘acceptor’ ubiquitin such that the α-amino group of its N-terminal methionine is available for conjugation to the distal ‘donor’ ubiquitin to form a linear linkage (Smit et al., 2012; Stieglitz et al., 2012; Stieglitz et al., 2013) (Figure 3B). Additionally, a zinc finger insert within the HOIP catalytic domain forms part of the acceptor ubiquitin binding platform and is important for chain synthesis. Like other E3 ligases, HOIP utilizes a conserved catalytic triad in which a conserved histidine proximal to the active-site cysteine acts as a general base to deprotonate the incoming acceptor ubiquitin nucleophile and mutation of this histidine, while permitting thioester formation, blocks aminolysis and renders the enzyme unable to form ubiquitin chains (Stieglitz et al., 2013; Lechtenberg et al., 2016). Because of its exquisitely specific catalytic mechanism it is highly likely that HOIP can only ubiquitylate pre-existing ubiquitin chains, something it has been shown to do in the context of innate immune signaling, where it assembles Met1-linked chains on existing Lys63-linked ubiquitin polymers (Emmerich et al., 2013; Emmerich et al., 2016).

As with ubiquitin chains assembled via conventional lysine linkages, Met1-linked ubiquitin chains are recognized by distinct ubiquitin binding proteins that act to translate the signal into specific cellular outcomes (Fennell et al., 2018). NEMO (NF-κB essential modifier) and ABIN1 (A20-binding inhibitor of NF-κB-1) play essential roles in the NF-κB signaling pathway by binding to Met1-linked ubiquitin chains via their UBAN domains (Ubiquitin-binding domain in ABIN and NEMO), a domain they also share with the autophagy receptor Optineurin (Wagner et al., 2008; Komander et al., 2009; Lo et al., 2009; Oshima et al., 2009; Rahighi et al., 2009; Nakazawa et al., 2016). Binding of Met1-linked ubiquitin chains to NEMO induces a conformational change in the protein that is necessary for activation of the IκB protein kinase complex and resulting downstream inflammatory signaling (Rahighi et al., 2009; Tokunaga et al., 2009; Kensche et al., 2012; Zhang et al., 2014; Hauenstein et al., 2017), whereas ABIN1 acts to restrict the signal, probably by competing with NEMO for ubiquitin chain binding (reviewed in Cohen and Strickson 2017). Met1-linked ubiquitin chains thus appear to act as molecular scaffolds to recruit and bind signaling complexes, as well as conventional second messengers that can activate the catalytic activity of those complexes.

Like many other regulatory post-translational modifications ubiquitylation is reversible, with ubiquitin chains existing in a dynamic equilibrium that balances E3-mediated assembly against enzyme-mediated disassembly by a family of approximately 100 deubiquitylases (DUBs) (Clague et al., 2019; Lange et al., 2022). Among the DUBs, Otulin (OTU deubiquitylase with linear linkage specificity) and CYLD (Cylindromatosis) are capable of hydrolyzing Met1-linked polyubiquitin selectively (Komander et al., 2009; Keusekotten et al., 2013; Rivkin et al., 2013; Ritorto et al., 2014; Sato et al., 2015). Interestingly, these DUBs associate with LUBAC by binding directly (Otulin) or indirectly (CYLD) to HOIP’s N-terminal PUB (PNGase/UBA or UBX-containing proteins) domain in a mutually exclusive manner (Elliott et al., 2014; Schaeffer et al., 2014; Takiuchi et al., 2014; Draber et al., 2015; Elliott et al., 2016; Kupka et al., 2016; Schlicher et al., 2016; Wagner et al., 2016). Mutations in the ‘writers’, ‘readers’, and ‘erasers’ of the Met1-linked ubiquitin signal are associated with various severe human pathologies, such as cancer, and inflammatory, autoimmune, and neurodegenerative diseases (Jahan et al., 2021), dramatically highlighting the importance of this atypical ubiquitin linkage.

### N-terminal ubiquitylation of other proteins

Well over a decade before the discovery of Met1-linked polyubiquitin chains, the lab of Alexander Varshavsky reported the intriguing finding that the N-terminal ubiquitin moiety of the artificial fusion protein Ubiquitin-Proline-β-galactosidase served as a degradation signal in yeast, permitting the formation of a polyubiquitin chain linked to Lys48 of the fused ubiquitin (Johnson et al., 1992). In this case the attachment of the ‘priming’ ubiquitin moiety was an engineered rather than a natural event, but N-terminal ubiquitylation was subsequently shown to occur naturally at the N-terminus of the mammalian transcription factor MyoD (Myoblast determination protein) (Breitschopf et al., 1998). A lysine-less MyoD mutant was still degraded *in vivo* and ubiquitylated forms of lysine-less MyoD accumulated after proteasomal inhibition. Moreover, selective chemical modification of the N-terminus or fusion of a Myc tag to the N-terminal residue of wild-type MyoD prevented degradation. Since then, similar biochemical approaches have been used to identify a number of other proteins degraded through N-terminal ubiquitylation, such as cyclin G1 (Li et al., 2009) and the cyclin-dependent kinase inhibitors p21^WAF/Cip1^ (Bloom et al., 2003; Coulombe et al., 2004), p19^ARF^ (Kuo et al., 2004), and p16^INK4a^ (Ben-Saadon et al., 2004; Kuo et al., 2004) amongst others (see McDowell and Philpott 2016 for more examples). While some direct evidence of N-terminal ubiquitylation was obtained in these early studies by mass spectrometry (Ben-Saadon et al., 2004; Coulombe et al., 2004), until recently no quantitative proteomics data was available on this modification. The global profiling of isopeptide-linked ubiquitylation sites was revolutionized a decade ago by the development of an antibody recognizing the diglycine remnant present on ubiquitylated lysines after tryptic digestion (Xu et al., 2010), fast becoming the method of choice for enrichment and identification of ubiquitylated sites (Kim et al., 2011; Udeshi et al., 2013; Steger et al., 2022). A major drawback of this technique however is that, due to the nature of the antibody epitope, it does not enrich for peptides originating from N-terminally ubiquitylated proteins. Recently other strategies, including the development of antibodies that recognize peptides bearing an N-terminal diglycine motif, have allowed detection of N-terminal ubiquitylation sites on a global scale (Akimov et al., 2018a; Akimov et al., 2018b; Davies et al., 2021; Trulsson et al., 2022). These studies reveal that the relative abundance of N-terminal ubiquitin linkages is exceedingly low (to date several hundred sites have been identified), likely due to the fact that 80–90% of human proteins can be acetylated at their N-termini, a modification that would preclude N-terminal ubiquitylation (Arnesen et al., 2009). Interestingly, no significant accumulation of N-terminal ubiquitylated proteins was observed upon proteasome inhibition, suggesting that N-terminal ubiquitylation has roles beyond that of a degradation signal.

**FIGURE 4:**
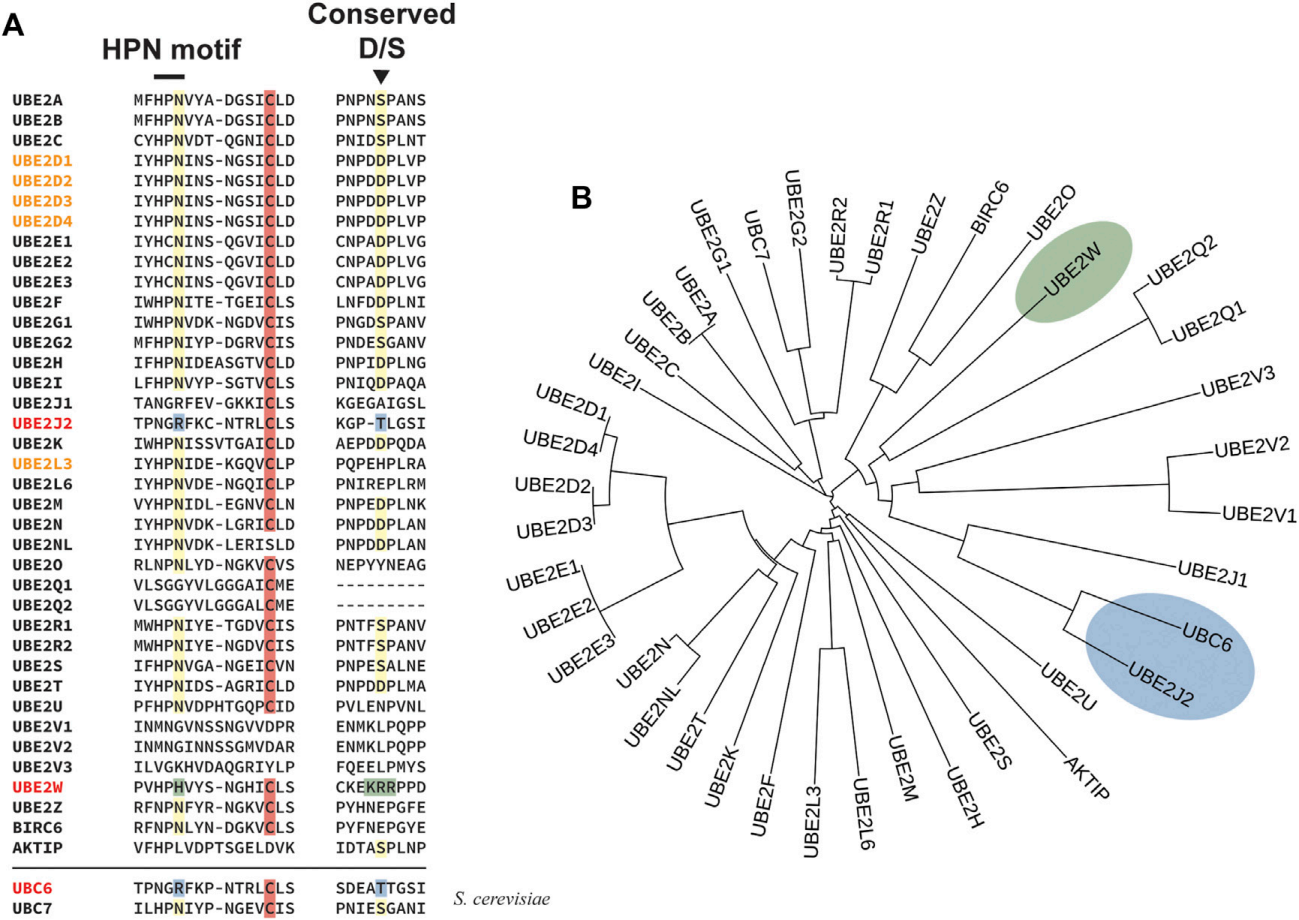
Novel sequence features of UBE2W and UBE2J2 that may determine non-canonical specificity. **(A)** A sequence alignment of the residues in selected human and yeast E2s close to the active site cysteine (highlighted in red) reveals that canonical lysine-directed E2s contain a conserved upstream arginine residue (normally part of an HPN motif) and a downstream Asp/Ser residue (both highlighted in yellow). E2s known to ubiquitylate hydroxylated residues are written in red and notably differ in the amino acids present at these sites. Divergent catalytically-important amino acids in UBE2W are highlighted in green, including the basic cluster that replaces the conserved D/S residue. The equivalent residues in UBE2J2 and its yeast homologue UBC6 are highlighted in blue. The names of E2s involved in transthiolation of cysteine residues are written in orange. Sequence alignments were performed using the Clustal Omega server (Sievers and Higgins 2021) and manually edited where necessary to match known structural information (Gundogdu and Walden 2019). **(B)** Phylogenetic tree depicting relationships between the selected E2s shown in **(A)**. The non-canonical ERAD-related E2 conjugating enzyme UBE2J2 and its yeast homologue UBC6 are highlighted in blue, UBE2W is highlighted in green. Analysis was performed by aligning the ubiquitin-conjugating UBC fold of selected E2s in Clustal Omega and is displayed using iTOL (Letunic and Bork 2021).

Thus far the ubiquitin conjugating enzyme UBE2W is the only E2 known to catalyze ubiquitylation of the N-termini of proteins (Scaglione et al., 2013; Tatham et al., 2013; Davies et al., 2021). UBE2W strictly monoubiquitylates its substrates, priming them for polyubiquitylation by other E2/E3 complexes. Unlike the HOIP subunit of LUBAC, UBE2W does not elongate linear ubiquitin chains, implying that the N-terminus of the attached monoubiquitin cannot serve as a UBE2W substrate. This is likely because ubiquitin’s N-terminus is highly structured and evidence suggests that UBE2W recognizes the peptide backbone of proteins that possess intrinsically disordered N-termini (Vittal et al., 2015). Comparison of the active site of UBE2W with those of classical E2s reveals distinct differences that make UBE2W better suited to accommodate an N-terminal α-amino group rather than the ε-amino group of a lysine residue side chain. Canonical E2s are characterized by a conserved His-Pro-Asn (HPN) motif where the critical asparagine residue (equivalent to N77 in UBE2D1) is thought to stabilize the oxyanion transition state intermediate (Wu et al., 2003) and play structural roles that facilitate transfer of ubiquitin to substrate lysines (Berndsen et al., 2013). Notably, UBE2W is unique among the ∼40 human E2s as it contains a histidine at this key position (Figure 4). Moreover, a highly conserved Asp/Ser residue thought to be important in canonical E2s for placement and deprotonation of the incoming substrate lysine (Yunus and Lima 2006; Plechanovova et al., 2012; Valimberti et al., 2015) is absent in UBE2W and replaced instead by a cluster of basic residues. A partly disordered and highly flexible C-terminus that can occupy multiple positions in proximity to the active site cysteine also allows UBE2W to accommodate a diverse set of disordered N-termini (Vittal et al., 2015). Despite these unusual features of UBE2W, it has still been reported to display some activity towards internal lysine residues in substrates (Scaglione et al., 2013; Fletcher et al., 2015) and recent proteomic analyses indicate that enzymes other than UBE2W are responsible for the majority of the N-terminal ubiquitylation sites reported to date (Davies et al., 2021), suggesting that other enzymes still await discovery. The E3 ubiquitin ligase HUWE1 (HECT, UBA and WWE domain containing 1) has been shown to ubiquitylate lysine-less MyoD on its N-terminus, but the physiological relevance of this is unclear as HUWE1 preferentially modifies internal lysines in MyoD and does not ubiquitylate the N-terminus of the wild-type protein (Noy et al., 2012).

Most N-terminal protein modifications, such as acetylation and initiator methionine removal, are irreversible events (Varland et al., 2015) but N-terminal ubiquitylation appears to be an exception. A family of enzymes known as the ubiquitin C-terminal hydrolases (UCHs) were the first class of DUB to be described (Rose and Warms 1983). Purified UCH enzymes are inactive against isopeptide- and Met1-linked ubiquitin dimers *in vitro* (Komander et al., 2009; Ritorto et al., 2014) but display deubiquitylase activity against N-terminally ubiquitylated peptides and various N-terminally ubiquitylated proteins, such as autoubiquitylated UBE2W (Woo et al., 1995; Bett et al., 2015). Interestingly, UCH family members are themselves substrates for UBE2W and ubiquitylation of their N-termini alters their DUB activity (Davies et al., 2021).

## Ubiquitylation of cysteine

**FIGURE 5:**
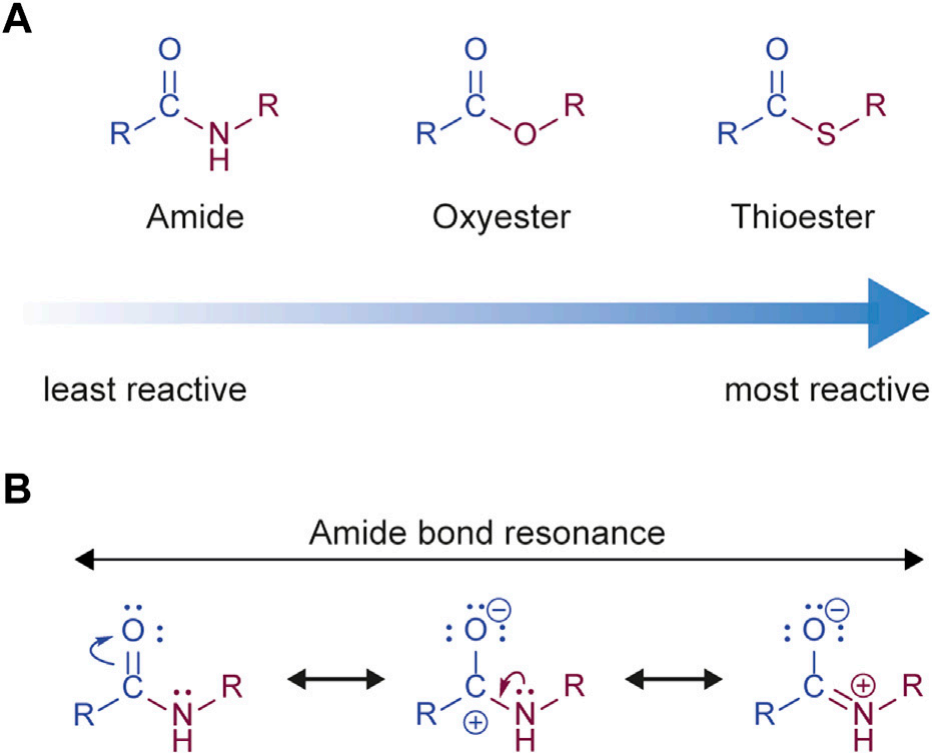
The relative reactivity of ubiquitin conjugating bonds. **(A)** The amide group is the least reactive, and therefore the most stable, of the acyl groups formed during ubiquitylation. The thioester is the most reactive, and therefore the least stable, whereas the oxyester is somewhere in between. **(B)** The stability of the amide bond is due to stabilization of the partial positive charge on the carbonyl carbon by electron donation from non-bonding electrons on the adjacent nitrogen, thus decreasing electrophilicity. In contrast, thioesters exhibit much weaker resonance stabilization due to poor overlap between the electron orbitals in sulfur and carbon.

Cysteines play a significant role in the ubiquitylation cascade: the E1 and E2 enzymes, as well as several classes of E3 enzyme, all bind to ubiquitin via thioester linkage to cysteine. Compared to amides (peptides/isopeptides), thioesters are significantly more reactive towards nucleophilic acyl transfer (Figure 5A). This is because the thioester bond is more strongly polarized than the amide, in which the partial positive charge on the carbonyl carbon is stabilized by electron donation from nonbinding electrons on the adjacent nitrogen, decreasing electrophilicity (Soderberg 2019) (Figure 5B). This, coupled with the fact that thiolate (RS^–^) is a good leaving group, means that the thioester linkage is more labile and has a lower thermodynamic stability than the amide bond, but also means that thioesters can be formed more quickly than amide bonds and are the more kinetically favoured reaction. Thioesters are therefore well-suited to processes requiring quick transient modification, such as the cysteine relays observed in the ubiquitylation cascade from E1 to E3. Intriguingly, evidence suggests that protein structure can stabilize biological thioesters, with the cellular half-life of a thioester-linked model substrate calculated to be several hours (Song et al., 2009). Therefore, in a biological context, the linkage is not as labile as might be imagined and ubiquitylation on cysteines could potentially provide a fast response to be employed in rapid signaling events which, coupled with the sensitivity of cysteines to the redox environment of the cell (Wang Y. et al., 2012) or even small changes in local pH (Marino and Gladyshev 2012), could provide a greater range of dynamic signaling behaviour than ubiquitylation on lysines alone. These concepts are nicely illustrated by the example of cysteine monoubiquitylation during protein import into peroxisomes.

### Cysteine ubiquitylation regulates peroxisomal protein import

**FIGURE 6:**
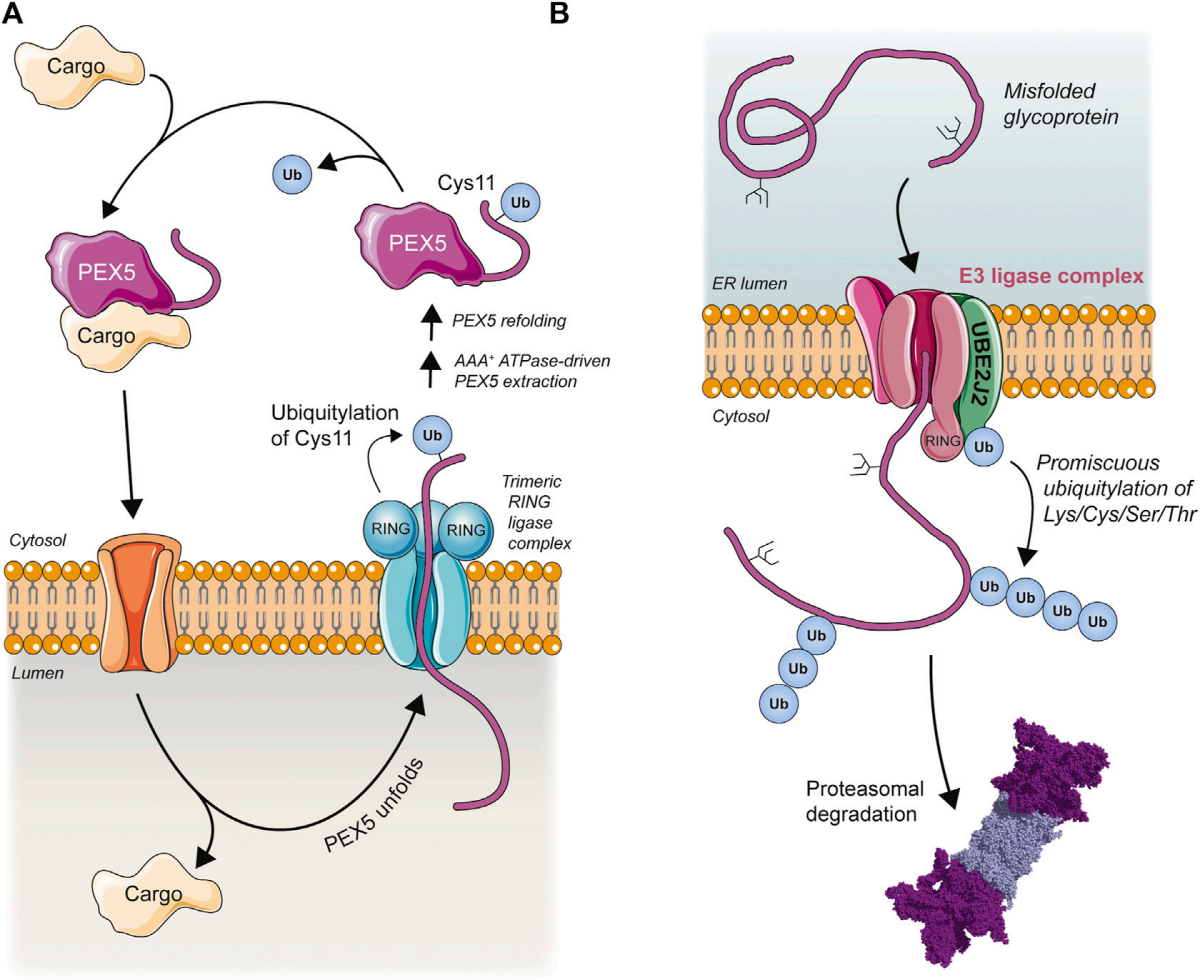
Membrane-spanning E3 ligase complexes facilitate non-lysine ubiquitylation during PEX5 recycling and ER-associated protein degradation. **(A)** Cargo proteins destined for peroxisomal import are recognized in the cytosol by PEX5. PEX5 and cargo then translocate from the cytosol into the lumen of the peroxisome. The unstructured N-terminus of PEX5 inserts into the pore of an E3 ligase complex containing three RING-type E3s and is monoubiquitylated on a cysteine residue, triggering the extraction of PEX5 from the peroxisome by a hexameric ATPase. This extraction is accompanied by PEX5 unfolding and cargo release. Once in the cytosol PEX5 refolds and is deubiquitylated, allowing a new round of cargo import to begin. **(B)** Misfolded or unfolded glycoproteins in the ER lumen are retrotranslocated into the cytosol by an E3 ligase complex and ubiquitylated by this complex upon emergence on the cytosolic side. The membrane-bound ubiquitin conjugating enzyme UBE2J2 acts with the ligase complex to modify a range of amino acid residues (Lys/Cys/Ser/Thr) in the misfolded target protein, triggering its proteasomal degradation.

Peroxisomes are highly versatile and dynamic organelles that play essential roles in cellular lipid metabolism, redox homeostasis, and innate immune signaling (Nordgren and Fransen 2014; Islinger et al., 2018; Di Cara et al., 2019). In contrast to other organelles, peroxisomes can import completely folded and even oligomeric or co-factor bound proteins into the peroxisomal matrix without prior unfolding of the protein (Glover et al., 1994; McNew and Goodman 1994; Titorenko et al., 2002). In humans and animals this protein import is mediated by the soluble cargo receptor PEX5 (Peroxin 5) (Walter and Erdmann 2019). PEX5 recognizes cytosolic cargo proteins bearing a peroxisomal targeting signal and transports them into the peroxisomal matrix via a transiently-formed and poorly-understood translocation channel (Francisco et al., 2017; Walter and Erdmann 2019; Skowyra and Rapoport 2022). Following cargo release into the peroxisomal matrix PEX5 is then extracted from peroxisomes by a hexameric AAA^+^ ATPase and recycled back to the cytosol for further rounds of import (Miyata and Fujiki 2005; Platta et al., 2005). Unusually, this recycling step requires PEX5 monoubiquitylation at a conserved cystine residue (Cys11) within the unstructured N-terminal region of the protein (Carvalho et al., 2007; Platta et al., 2007; Williams et al., 2007; Okumoto et al., 2011) (Figure 6A). Replacing Cys11 with lysine still permits PEX5 recycling but results in polyubiquitylation that increases the likelihood of PEX5 degradation rather than recycling (Grou et al., 2009; Schwartzkopff et al., 2015). Related proteins in *Saccharomyces cerevisiae* and *Pichia pastoris* are similarly released into the cytosol by a process that requires cysteine-dependent monoubiquitylation (El Magraoui et al., 2013; Liu and Subramani 2013) and loss of functional protein import/export occurs upon mutation of the corresponding N-terminal cysteine residues to serine or alanine (Leon and Subramani 2007; Hensel et al., 2011; Rudowitz et al., 2020).

A complex of three membrane-embedded RING-type E3 ubiquitin ligases (PEX2, PEX10, and PEX12) functions to ubiquitylate PEX5 (Williams et al., 2008; Platta et al., 2009; El Magraoui et al., 2012; Okumoto et al., 2014). The three subunits form a channel with an open ∼10 Å pore in which the RING domains are positioned on the cytosolic side ready to ubiquitylate the emerging PEX5 (Feng et al., 2022). In yeast, Pex5 ubiquitylation is catalyzed by the membrane-tethered E2 enzyme Pex4 (Wiebel and Kunau 1992; Platta et al., 2007; Williams et al., 2007; El Magraoui et al., 2014) whereas in humans the E2 enzymes UBE2D1, UBE2D2, and UBE2D3 have been proposed to function with the peroxisomal E3 ligase complex to catalyze Cys11 monoubiquitylation of PEX5 (Grou et al., 2008). In addition to its monoubiquitylation on cysteine, PEX5 can also be mono- and polyubiquitylated on lysine residues by the PEX2/PEX10/PEX12 ligase complex, as well as other ligases such as TRIM37 (Tripartite Motif Containing 37) in order to regulate PEX5 stability (reviewed in Wang and Subramani 2017). In contrast to UBE2W discussed earlier, the E2s reported to catalyze cysteine ubiquitylation of PEX5 are not specifically adapted (Figure 4A); indeed, as members of the UBE2D family they represent the very definition of a ‘canonical’ E2, also mediating the conventional lysine-directed ubiquitylation of PEX5 (Platta et al., 2004; Wang W. et al., 2017). These E2s are, however, extremely promiscuous (Stewart et al., 2016) and have been reported to possess an intrinsic E3-independent reactivity against both lysine and cysteine residues (Wenzel et al., 2011). Indeed, the ability to replace Cys11 with lysine and still achieve ubiquitylation (Grou et al., 2009; Schwartzkopff et al., 2015) suggests that, from an E2/E3 point-of-view, it is the position of the ubiquitylation site that is important, not necessarily the residue. So why is cystine preferred?

As already detailed, the intrinsic lability of thioesters makes them well-suited to transient responses such as rapid PEX5 recycling. In addition, the nucleophilicity of the cysteine residue is sensitive to changes in both redox environment and local pH (Pace and Weerapana 2013). As suggested by their name, peroxisomes contain enzymes that both produce and degrade hydrogen peroxide (De Duve and Baudhuin 1966) as well as several other reactive oxygen species (Nordgren and Fransen 2014). Studies from both yeast and mammalian cells suggest that monoubiquitylation on cysteine serves as a redox-sensitive switch, allowing cells to sense and cope with redox stress by regulating PEX5 ubiquitylation and PEX5-mediated protein import in response (Ma et al., 2013; Apanasets et al., 2014; Walton et al., 2017). Under oxidizing conditions PEX5 cannot be monoubiquitylated at Cys11 and is retained in the translocation machinery (Apanasets et al., 2014). This prevents the import of the antioxidant enzyme catalase into peroxisomes, causing its retention in the cytosol where it can protect the cell against oxidative damage in a manner that peroxisomally-targeted catalase cannot (Walton et al., 2017). By contrast, oxidizing conditions only weakly decrease the monoubiquitylation of a C11K mutant of PEX5 (Apanasets et al., 2014), with the decreased ubiquitylation likely due to the effects of oxidation on the cysteine-containing enzymes of the ubiquitylation cascade (Jahngen-Hodge et al., 1997). Therefore, cysteine ubiquitylation extends the biological possibilities of ubiquitylation beyond those afforded by lysine, offering new functions and methods for regulation.

The PEX5 import cycle is completed by deubiquitylation in the cytosol (Figure 6A) (El Magraoui et al., 2019). Non-enzymatic cleavage by the major cytosolic nucleophile glutathione has been proposed (Grou et al., 2009) but biochemical studies in yeast and mammals have identified the PEX5-directed DUBs Ubp15 (Debelyy et al., 2011) and USP9X (Grou et al., 2012), respectively. It is possible that additional or redundant DUBs may also be involved in PEX5 deubiquitylation at Cys11 as many DUBs possess significant cysteine thioesterase activity (De Cesare et al., 2021) and recently a pool of USP30 has been reported to localize to peroxisomes in cells (Marcassa et al., 2018; Riccio et al., 2019).

In addition to PEX5, several other proteins have been reported to be ubiquitylated on cysteine residues (see McClellan et al., 2019 for further examples). Of note, studies on proteins regulating lipid homeostasis have revealed further examples of redox regulated cysteine ubiquitylation. Reactive oxygen species generated during lipid-overload-induced oxidative stress competitively block the cysteine-directed ubiquitylation of ACAT2 (Acyl-coA:cholesterol acyltransferase 2) and INSIG2 (Insulin-induced gene 2) by competitively oxidizing the modified cysteine residue, leading to stabilization of these proteins and a resulting decrease in cellular lipid levels that can ameliorate lipotoxicity (Wang YJ. et al., 2017; Zhou et al., 2020). It is possible that competitive oxidation and ubiquitylation on cysteine may be a common mechanism regulating many proteins in response to oxidative stress, but since most experimental samples are prepared in the presence of strong reducing agents that are not conducive to investigation of thiol-sensitive ubiquitylation, the true extent of cysteine ubiquitylation is currently unknown.

## Ubiquitin-oxyester linkages

Ubiquitin can also be attached to serines, threonines, and (theoretically) tyrosines via an oxyester linkage (Figure 2C). This bond is more stable than a thioester, but less stable than an amide linkage (Figure 5A), with a reported cellular half-life of several hours at 37°C (De Cesare et al., 2021). While there are confirmed reports of serine and threonine forming ubiquitin-oxyester linkages, there are as yet no reports of tyrosines being ubiquitylated in this way. Due to the delocalization of electrons in its aromatic ring, tyrosine is less reactive than the aliphatic residues serine and threonine, but given that tyrosines are the target of other post-translational modifications (Jones et al., 2014) their ubiquitylation should not be ruled out.

### ERAD and UBE2J2

The earliest evidence for non-amide-linked ubiquitylation came from studies on the viral MARCH (membrane-associated RING-CH) ligase known as kK3 (Kaposi’s sarcoma-associated Herpesvirus protein K3), which was shown to promote the degradation of major histocompatibility complex (MHC) class 1 heavy chain by ubiquitylating it on a conserved cysteine residue in its cytoplasmic tail (Cadwell and Coscoy 2005). Just as surprising as this initial discovery of thioester-linked ubiquitylation was the finding that mK3, a homologue of kK3 expressed by murine ɣ-herpesvirus-68, promoted MHC-1 HC degradation via ubiquitylation of serine or threonine residues, and not cysteine (Wang et al., 2007), leading to the formation of a base-labile oxyester bond rather than a reductant-sensitive thioester and providing the first evidence for such a linkage in a biological system.

In both these examples the viral ligases exploit a cellular pathway known as endoplasmic reticulum (ER)-associated degradation (ERAD) to downregulate MHC-1 expression and evade immune recognition (Byun et al., 2014). A multi-step pathway involving the chaperone-mediated selection of misfolded proteins in the ER and their retrotranslocation through the ER membrane for ubiquitylation and degradation in the cytosol (Figure 6B) (Lemberg and Strisovsky 2021), to date ERAD has provided more examples of oxyester-linked ubiquitylation than any other biological process (see McClellan et al., 2019). Surprisingly, despite the structural diversity and sheer quantity of potential ERAD substrates (estimated to be up to one-third of all eukaryotic proteins (Huh et al., 2003)), only a handful of E2s and E3s have been implicated in ERAD (Christianson and Ye 2014), suggesting that the ERAD ubiquitylation machinery operates more opportunistically and with less substrate stringency than the cytoplasmic quality control system. This is consistent with data that reveals a promiscuous ubiquitylation of lysine, cysteine, serine, and threonine residues in ERAD substrates (Ishikura et al., 2010; Shimizu et al., 2010; Burr et al., 2013; Boban et al., 2015; Weber et al., 2016; Chua et al., 2019).

Yeast ERAD relies mainly on two E2 enzymes one of which, Ubc6, has been shown to target the hydroxylated amino acids serine and threonine (Weber et al., 2016). The human orthologue of Ubc6 is the ER-membrane-associated protein UBE2J2, which has been shown to preferentially promote ubiquitylation of hydroxylated amino acids, even when lysine residues are present on substrates (Wang et al., 2009; De Cesare et al., 2021). Similar to the situation described earlier for PEX5 ubiquitylation, there is target residue flexibility in ERAD—ubiquitylated serine and threonine residues may be experimentally substituted for lysine or cysteine residues with little effect on ubiquitylation efficiency and substrate degradation—suggesting that the essential determinant driving substrate ubiquitylation may be target residue position rather than identity (Cadwell and Coscoy 2005; Wang et al., 2007; Cadwell and Coscoy 2008; Herr et al., 2009; Wang et al., 2009; Ishikura et al., 2010). And like UBE2W, UBE2J2 lacks the canonical HPN motif involved in catalysis, as well as the nearby Asp/Ser residue thought to be involved in suppressing the p*K*_a_ of substrate lysine residues during canonical isopeptide bond formation (Figure 4A) suggesting that the absence of these motifs might be a common theme in enzymes targeting residues other than lysine. A further notable parallel between PEX5 recycling, UBE2W-catalysed ubiquitylation and UBE2J2 activity is that UBE2J2 is primarily a monoubiquitylating enzyme (De Cesare et al., 2021) whose yeast homologue, Ubc6, requires the activity of another E2, Ubc7 (homologue of human UBE2G2), in order to extend the initial Ser/Thr ubiquitylation and polyubiquitylate substrate proteins with K48-linked ubiquitin chains, targeting them for proteasomal degradation (Weber et al., 2016; Lips et al., 2020; Schmidt et al., 2020). In an interesting twist Ubc7 itself can be degraded by polyubiquitylation of its active site cysteine by the HECT-domain ligase Ufd4 (Ravid and Hochstrasser 2007).

Budding yeast rely upon two non-redundant RING type E3 ubiquitin ligases, operating in conjunction with E2s Ubc6 and Ubc7, to modify substrates with K48-linked polyubiquitin chains (Vashist and Ng 2004; Carvalho et al., 2006). Both of these ligases, Doa10 and Hrd1, are evolutionarily conserved, but humans also use an expanded collection of ERAD-related RING and U-box E3s (Christianson and Ye 2014). There is currently little evidence that, beyond HRD1 and MARCH6 (the human Doa10 homologue), any of these additional ligases partner with UBE2J2 to catalyze non-lysine ubiquitylation under normal conditions, but it has been speculated that during cytomegalovirus infection the ligase TMEM129, which uses UBE2J2 as its cognate E2 partner, is appropriated by the viral protein US11 to degrade MHC-1 HC via oxyester-linked ubiquitylation (van den Boomen et al., 2014; McClellan et al., 2019). This remains to be directly determined.

### HOIL-1

Before it was identified as a component of LUBAC, HOIL-1 was independently cloned and variously described as a binding partner and E3 ubiquitin ligase for oxidized iron regulatory protein 2 (IRP2) (Yamanaka et al., 2003), a protein associated with hepatitis B virus X protein (Cong et al., 1997), a binding partner of protein kinase C (PKC) (Tokunaga et al., 1998), and a specific interactor with the ubiquitin conjugating enzyme UBE2L3 (Martinez-Noel et al., 1999). Early work characterized HOIL-1 as a K48-specific ubiquitin ligase (Yamanaka et al., 2003; Bayle et al., 2006; Zenke-Kawasaki et al., 2007; Zhang et al., 2008; Rana et al., 2013; Queisser et al., 2014) whereas an *in vitro* study from 2012 found HOIL-1 to possess an extremely weak E3 ligase activity that synthesized purely Met1-linked ubiquitin chains (Stieglitz et al., 2012). In disagreement with these reports a much overlooked study from 2008 came to the conclusion that HOIL-1 could autoubiquitylate *in vitro* with a strong preference for monoubiquitylation (Tatematsu et al., 2008) and a further study reported that HOIP and HOIL-1 were both required for the polyubiquitylation of NEMO *in vitro* (Smit et al., 2013).

Recently the characterization of catalytically inactive HOIL-1 knock-in mice combined with biochemical assays that tested for non-lysine ubiquitylation revealed that HOIL-1 catalyzes the ubiquitylation of serine and threonine on several physiological substrates *in vivo*, including itself, fellow LUBAC component Sharpin, the IL-1 receptor associated kinases IRAK1 and IRAK2, and the Toll-like receptor (TLR) adapter protein MyD88 (Myeloid differentiation factor 88), all of which are components of, or proteins associated with, a large oligomeric immune signaling complex known as the Myddosome (Kelsall et al., 2019). Together with further work by other labs, this study confirmed that HOIL-1 acts to conjugate monoubiquitin onto its substrates via an oxyester linkage, permitting subsequent chain elongation by other ligases, such as the Met1-specific E3 ligase HOIP (Kelsall et al., 2019; Fuseya et al., 2020; Petrova et al., 2021; Rodriguez Carvajal et al., 2021). A complex and dynamic interplay between oxyester-linked and amide-linked ubiquitylation operates during immune receptor stimulation, with oxyester-anchored chains apparently acting to limit the size and number of conventional isopeptide-anchored and amide-linked chains on substrates (Kelsall et al., 2019; Petrova et al., 2021). Furthermore, HOIL-1 can monoubiquitylate HOIP, allowing the conjugation of linear ubiquitin chains to HOIP that attenuate the Met1-linked ubiquitylation of other LUBAC targets in cells (Heger et al., 2018; Fuseya et al., 2020). This interplay between amide-linked and oxyester-linked ubiquitylation is also highlighted by a recent report that Met1-linked ubiquitin chains produced by HOIP—and K63-linked ubiquitin chains produced by ligases such as TRAF6 (TNF receptor-associated factor 6) (Deng et al., 2000)—allosterically activate HOIL-1 catalytic activity *in vitro* (Kelsall et al., 2022). *In vivo*, ester-linked ubiquitin tunes the strength of the immune signal and may therefore promote or inhibit cytokine production in immune cells depending on the nature of the stimuli and the type of cell analyzed (Petrova et al., 2021).

**FIGURE 7:**
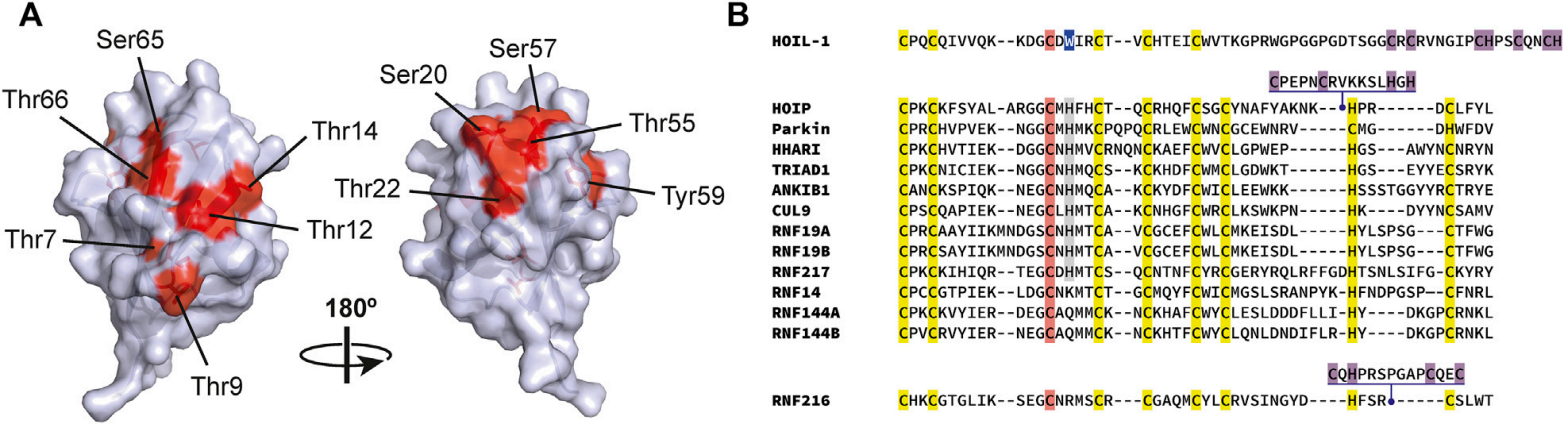
HOIL-1 catalyzes serine and threonine ubiquitylation. **(A)** Structure of ubiquitin (PDB: 1UBQ) (Vijay-Kumar et al., 1987) highlighting the eleven hydroxylated amino acids (shown in red). HOIL-1 can form ubiquitin dimers *in vitro*, with oxyester linkages at Thr12, Ser20, Thr22, and Thr55 all reported. Note that the last three of these residues cluster at a single region on the surface of ubiquitin. **(B)** Alignment of the human RBR domain sequences in the catalytic RING2 domain. Conserved zinc coordinating residues that form the RING2 fold are highlighted yellow, the active site cysteine is highlighted in red, the catalytic histidine residue present in most RBRs is highlighted in gray; in HOIL-1 this is a tryptophan and is highlighted in blue. Unique inserts exist in HOIL-1, HOIP, and RNF216, and putative or confirmed zinc coordinating residues are highlighted purple. The identity of the last three zinc binding residues in the HOIL-1 RING domain is currently unknown and consequently these residues are not aligned.

Intriguingly, HOIL-1 can form oxyester linkages between ubiquitin monomers *in vitro*, with oxyester linkages at Thr12, Ser20, Thr22 and Thr55 all identified by mass spectrometry (Kelsall et al., 2019; Rodriguez Carvajal et al., 2021). Indeed, Rodriguez Carvajal et al. (2021) reconstituted a highly pure LUBAC holoenzyme by co-expression of the LUBAC components in insect cells and found that the complex assembled highly branched linear ubiquitin chains in which branching was achieved by oxyester bond formation between ubiquitin molecules. These oxyester linkages within ubiquitin chains may also act to limit or terminate chain extension, possibly explaining why the ubiquitin chains that become attached to IRAK1 and IRAK2 during TLR signaling are much larger in macrophages from mice expressing an E3 ligase-inactive mutant of HOIL-1 (Kelsall et al., 2019). To date, the existence of oxyester-linked ubiquitin dimers has not yet been directly confirmed by mass spectrometry *in vivo*, but it is notable that ubiquitin—which contains seven threonine residues, three serine residues, and one tyrosine—possesses a greater number of hydroxylated residues capable of forming oxyester-linked chains than it does lysine residues (Figure 7A). The potential therefore exists for a significant expansion of the existing ubiquitin code.

No high-resolution structure is available for the catalytic domain of HOIL-1. As a transthiolating RBR ligase HOIL-1 is thought to determine target specificity (rather than its cognate E2) and so structural information will be crucial to understand how HOIL-1 achieves specificity, but crucial also to explain the inherent flexibility in HOIL-1 activity that permits it to catalyze both oxyester- and, some degree, isopeptide-linked ubiquitylation *in vitro* (Fuseya et al., 2020). In many other RBR ligases, such as HOIP or HHARI, a conserved histidine residue close to the catalytic cystine acts as a general base to deprotonate the incoming acceptor ubiquitin nucleophile (Cotton and Lechtenberg 2020). Uniquely amongst the RBR ligases, HOIL-1 contains a tryptophan residue at this position (Figure 7B), suggestive of a specifically tailored catalytic mechanism. It is notable that HOIL-1 also contains an unusual extension in its catalytic domain consisting of a number of potential zinc binding residues. The RBR ligases HOIP and RNF216 both contain zinc-finger insertions in their catalytic domains that help dictate their distinct chain-type specificities (Stieglitz et al., 2013; Cotton et al., 2022) and so the presence of an additional zinc binding site in HOIL-1 raises the possibility that this may provide the unique functionality permitting its formation of oxyester linkages. It has also been suggested, based upon cross-linking mass spectrometry and a low-resolution 3D reconstruction of the human complex, that LUBAC contains a single catalytic core made up of the catalytic domains of both HOIL-1 and HOIP (Rodriguez Carvajal et al., 2021), raising the possibility that LUBAC may utilize a coordinated ubiquitin relay mechanism, similar to that observed in the RCR ligase MYCBP2 (Pao et al., 2018).

### MYCBP2

The transient nature of protein-protein interactions, combined with the lability of the thioester bond, complexity of the ubiquitylation cascade, and the remarkable speed with which ubiquitylation reactions can occur (Pierce et al., 2009), have led to the development of a range of chemical biological tools to stabilize the fleeting transition states that exist (Henneberg and Schulman 2021). The use of one such ‘activity-based probe’ unexpectedly identified the E3 ligase MYCBP2 (Myc-binding protein 2) as a transthiolating enzyme in possession of an unprecedented 30 kDa enzymatic RCR (RING-Cys-Relay) module consisting of a RING domain followed by two downstream catalytic cysteine residues residing within a unique zinc-binding domain (Pao et al., 2018). The first of these cysteines receives ubiquitin from the catalytic cysteine of the RING-bound E2 UBE2D3 and ‘relays’ it intramolecularly over a distance of approximately 24 Å to the second cysteine (Pao et al., 2018; Mabbitt et al., 2020). This second site utilizes a canonical catalytic triad of cysteine, histidine, and glutamic acid to transfer the ubiquitin to substrates. Strikingly, MYCBP2 has no activity against lysine and instead targets the hydroxylated residues threonine and serine, with a strong preference observed for threonine in model substrates. This threonine selectivity may be understood from the crystal structure, which reveals that the esterification site of the enzyme contains a hydrophobic pocket that engages the side chain methyl group of threonine and optimally positions the substrate hydroxyl for catalysis (Pao et al., 2018).

MYCBP2 plays an important role in the programmed degeneration of nerve fibres following injury (Virdee 2022). Mutations that disrupt the RING domain of MYCBP2 in *Drosophila* stabilize the axon survival factor NMNAT2 (Nicotinamide mononucleotide adenylyltransferase 2), one of the most likely targets of MYCBP2’s non-lysine E3 activity (Xiong et al., 2012). MYCBP2 has been shown to ubiquitylate NMNAT2 by esterification *in vitro* (Pao et al., 2018) and knock-in mice in which the catalytic relay of MYCBP2 is disrupted by mutation display delayed injury-induced axon degeneration (Mabbitt et al., 2020). These results highlight the important role that oxyester-linked ubiquitylation may play during pathological neuropathies and neurodegenerative disorders such as Parkinson disease, where programmed axon degeneration plays a prominent role (Adalbert and Coleman 2013).

### Deubiquitylases with activity against oxyester linkages in representative model substrates

Around two-thirds of known DUBs have been screened for threonine esterase activity, leading to the surprising discovery that most DUBs are almost equally capable of cleaving both isopeptide- and oxyester-linked ubiquitin from substrates (Sun et al., 2018; De Cesare et al., 2021). Notable exceptions are the OTU (Ovarian Tumour Domain) family of DUBs that demonstrate negligible threonine esterase activity against model substrates, consistent with the specialization of this class of DUBs for cleavage of specific isopeptide-linked polyubiquitin chains (Mevissen et al., 2013). Members of the MJD (Machado-Josephine Disease) family of DUBs, on the other hand, display a selective esterase activity toward both serine and threonine that appears to be facilitated by the conspicuously hydrophobic nature of their catalytic sites (De Cesare et al., 2021). To date only a small number of arguably non-physiological substrates have been analyzed, but the ability of so many DUBs to deubiquitylate oxyester-linked substrates, and of some DUBs to do so preferentially, suggests that non-lysine ubiquitylation, far from representing a minor cellular peculiarity, may in fact occur on a scale not previously imagined.

## Non-protein substrates

Until recently only proteins were considered as substrates for ubiquitylation. This focus on proteins is understandable given that ubiquitylation was originally discovered as a protein degradation signal (see Ciechanover 2009 for a historical perspective) but fails to account for the catalytic pliancy of the ubiquitylation machinery, which has often proved itself capable of working with a range of small non-protein substrates *in vitro* (Rose and Warms 1983; Pickart and Rose 1985a; Pickart and Rose 1985b; Wenzel et al., 2011; Pao et al., 2018; Lips et al., 2020). It has been known for over 2 decades that a ubiquitin-like conjugation reaction can form an amide bond between members of the Atg8 family of ubiquitin-like proteins and membrane lipids such as phosphatidylethanolamine (Ichimura et al., 2000) but whether such non-proteinaceous substrates existed for ubiquitin was, until recently, completely unknown. Now several studies published within the last 5 years have uncovered a range of non-protein substrates that includes ADP-ribose, bacterial lipopolysaccharide (LPS), and cellular sugars.

### RNF213 and lipopolysaccharide ubiquitylation

**FIGURE 8:**
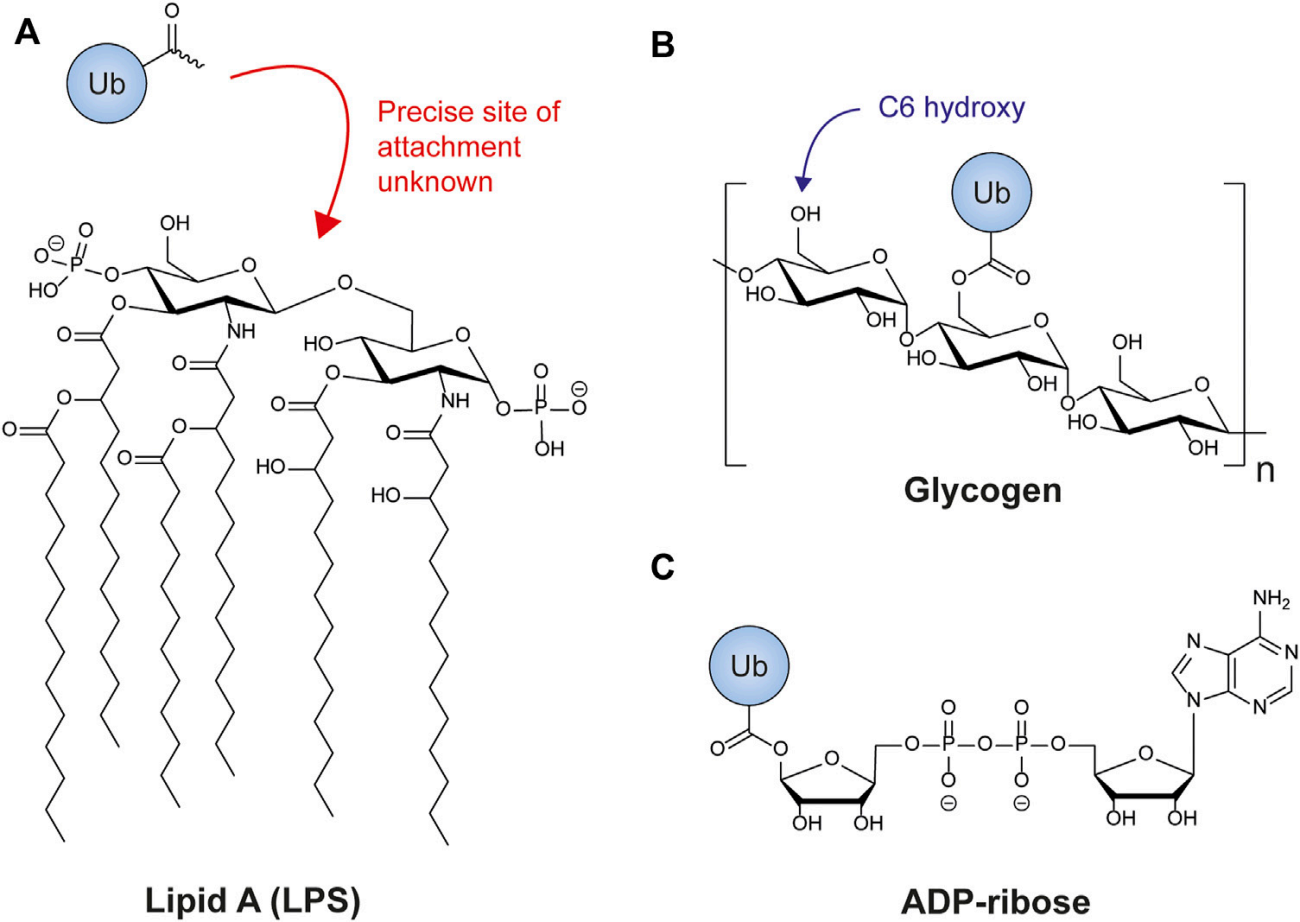
Non-proteinaceous substrates of ubiquitylation. **(A)** The Lipid A component of bacterial lipopolysaccharide represents the minimal substrate for RNF213-mediated ubiquitylation of LPS. The precise site of attachment is not yet known. **(B)** Glycogen is a highly branched polymer of glucose. Most glucose units are linked together by α(1,4)-glycosidic bonds with branches formed by the introduction of glycosidic bonds at the C6 hydroxy position every 8–12 glucose units. This C6 hydroxy group is also the target of HOIL-1-catalysed ubiquitylation of glycogen and related sugars. **(C)** The Deltex family E3 ligases can catalyze the conjugation of ubiquitin to ADP-ribose by means of an oxyester linkage between the 1′-hydroxy group of ribose and ubiquitin’s C-terminus. This reaction requires all the components of the ubiquitylation cascade and involves no recognized ADP-ribosyltransferase.

Ubiquitin plays an important part in the host cell’s defense against bacterial infection (reviewed in Tripathi-Giesgen et al., 2021). Invading bacteria are labelled with a coating of ubiquitin that activates subsequent antibacterial autophagy (Perrin et al., 2004). The ubiquitylation of several bacterial outer membrane proteins has been reported (Fiskin et al., 2016) but recently Otten *et al.* (2021) made the unexpected discovery that the outer membrane glycolipid LPS could be ubiquitylated in the Gram-negative bacteria *Salmonella enterica*. Using *Salmonella* mutants defective in generating portions of LPS, the authors could show that hydroxyl groups in the Lipid A component of LPS are the targets of ubiquitylation (Figure 8A). Biochemical fractionation from cell lysates led to the identification of RNF213 as the enzyme responsible for this unusual activity (Otten et al., 2021). This giant, multi-domain E3 ligase is the largest single-chain E3 identified in the human proteome and mutations in the gene encoding it are associated with the progressive cerebrovascular disorder Moyamoya disease (Kamada et al., 2011; Liu et al., 2011). Surprisingly, the RING domain of RNF213 is not required for its intrinsic E3 ligase activity (Ahel et al., 2020), nor its ubiquitylation of LPS (Otten et al., 2021). Instead, an adjacent zinc-binding domain uses a cysteine-dependent transthiolation mechanism to conjugate ubiquitin in a manner reminiscent of the HECT, RBR, and RCR families of E3 ligases (Ahel et al., 2021; Otten et al., 2021).

A cascade of E3 enzymes generates and shapes the ubiquitin coat associated with cytosolic bacteria (Tripathi-Giesgen et al., 2021). Of these, LUBAC requires pre-existing ubiquitylation of invading bacteria for its own recruitment (Noad et al., 2017; van Wijk et al., 2017). Otten *et al.* (2021) found that cells lacking RNF213, or containing mutations in RNF213s catalytic zinc-binding domain, failed to recruit LUBAC and to accumulate Met1-linked ubiquitin chains on the bacterial cell surface, indicating that RNF213-mediated LPS ubiquitylation functions to initiate LUBAC-mediated immune signaling. Intriguingly, RNF213 has also been reported to counteract infection with the Gram-positive bacterium *Listeria monocytogenes*, which lacks LPS (Thery et al., 2021), implying that other targets of RNF213 activity exist. It is notable too that mutations in RNF213 that predispose individuals to Moyamoya disease do not affect LPS ubiquitylation (Otten et al., 2021) suggesting that bacterial ubiquitylation does not play a role in the disease, which may instead be caused by other functional defects in RNF213.

Consistent with the promiscuous esterase activity displayed by many DUBs (De Cesare et al., 2021), the ubiquitylation of LPS was found to be antagonized by the broad-specificity deubiquitylase USP2 *in vitro* (Otten et al., 2021). Whether further DUBs, either host- or *Salmonella*-derived, can catalyze LPS deubiquitylation remains an open question. It is tempting to speculate that the ubiquitylation of non-proteinaceous substrates might represent a general mechanism for the detection and clearance of many different kinds of invading pathogen. Equally intriguing is the thought that other non-proteinaceous biomolecules, such as host cell lipids, might serve as targets of RNF213 ubiquitylation. Studies linking RNF213 to lipid metabolism (Piccolis et al., 2019) and lipid drop formation (Sugihara et al., 2019) make this an idea worthy of further investigation.

### HOIL-1 and polysaccharide ubiquitylation

In addition to its ability to ubiquitylate serine and threonine residues in substrates, the LUBAC component HOIL-1 is also capable of ubiquitylating non-protein substrates (Kelsall et al., 2022). Human patients deficient in HOIL-1 accumulate dense inclusions of insoluble starch-like polysaccharide in a number of organs including skeletal and cardiac muscle, resulting in cardiomyopathy and heart failure necessitating cardiac transplant (Boisson et al., 2012; Nilsson et al., 2013; Wang et al., 2013; Fanin et al., 2015; Krenn et al., 2018; Phadke et al., 2020; Chen et al., 2021; AlAnzi et al., 2022; Krishnan et al., 2022). These precipitates, known as Polyglucosan Bodies, are also observed in the heart and brain of mice expressing catalytically inactive HOIL-1, establishing that the E3 ligase activity of the enzyme is required to prevent polyglucosan accumulation (Kelsall et al., 2022). Polyglucosan represents an aberrant poorly-branched form of the normally high-branched glucose storage polymer glycogen. Tellingly, the HOIL-1 interacting proteins HOIP and Sharpin both bind to polyglucosan-like polysaccharides *in vitro* but fail to bind to healthy glycogen, suggesting that LUBAC may localize to sites of polyglucosan deposition in cells. *In vitro* ubiquitylation experiments have revealed that HOIL-1 can directly monoubiquitylate glycogen and smaller model oligosaccharides via an oxyester linkage. Interestingly the site of this ubiquitylation is the C6-hydroxy moiety of the hydroxymethyl group of the glucose monomers that make up glycogen (Kelsall et al., 2022) (Figure 8B), the same hydroxy group that is used to form a branch point in normal soluble glycogen (Wolfrom and O'Neill 1949; Cori and Larner 1951). This has led to speculation that HOIL-1 may act as part of a quality control mechanism to ubiquitylate and thereby promote the removal of erroneously formed and insufficiently branched glycogen prior to its precipitation from cells as polyglucosan bodies (Kelsall et al., 2022).

While interesting, it should be stressed that these studies represent *in vitro* results only and further work is still needed to establish the link between HOIL-1’s monoubiquitylation of oligosaccharides *in vitro* and the essential role played by HOIL-1’s E3 ligase activity in preventing polyglucosan deposition *in vivo*. In particular there is, as yet, no evidence of glycogen ubiquitylation *in vivo*. Nor is there any evidence that LUBAC associates with polyglucosan deposits in cells. Several inherited diseases of glycogen metabolism are characterized by polyglucosan body deposition (Hedberg-Oldfors and Oldfors 2015). One of these, Lafora Disease, is reminiscent of HOIL-1 deficiency and is also caused by mutations in genes not encoding the classical enzymes of glycogen synthesis. Notably, both Lafora Disease and HOIL-1 deficiency lead to hyperphosphorylation of glycogen at the C6 hydroxy group, something not observed in adult polyglucosan body disease caused by mutations in GBE1 (Glycogen Branching Enzyme 1) (Tagliabracci et al., 2008; Turnbull et al., 2010; DePaoli-Roach et al., 2015; Nitschke et al., 2017; Sullivan et al., 2019; Nitschke et al., 2022). This observation reveals an intriguing interplay between glycogen branching, ubiquitylation, and phosphorylation at the C6 position while also suggesting that HOIL-1 may operate as part of a system that intersects or parallels that which malfunctions to cause Lafora Disease. Approximately 50% of Lafora Disease patients have mutations in the gene encoding a RING-family E3 ubiquitin ligase called Malin (Mitra et al., 2022). Previous studies have assumed the physiological substrates of Malin to be proteins participating in glycogen synthesis and regulation (Gentry et al., 2005; Cheng et al., 2007; Vilchez et al., 2007; Solaz-Fuster et al., 2008; Worby et al., 2008; Moreno et al., 2010; Rubio-Villena et al., 2013) but in light of the priming role observed for HOIL-1 in ubiquitylation of the proteins of the Myddosome an attractive alternative is that Malin may act in concert with HOIL-1 to extend HOIL-1’s initial priming monoubiquitin and form polyubiquitin chains on glycogen. HOIL-1’s ability to ubiquitylate sugars also makes the involvement of LUBAC in the ubiquitylation of cytosolic bacteria such as *Salmonella* an interesting avenue for further study. Can HOIL-1 ubiquitylate LPS, like RNF213, or are there other non-protein bacterial substrates for HOIL-1 activity such as peptidoglycan? The sequential recruitment of two ligases capable of ubiquitylating non-protein substrates is a highly unexpected development in our understanding of cell-autonomous immunity and one that opens up an exciting new world of possibilities and opportunities.

### Deltex ligases and ADP-ribose

There are now multiple examples of interplay between ubiquitylation and ADP-ribosylation, a post-translational modification that reversibly attaches one or multiple adenosine 5′-diphosphate (ADP)-ribose units onto substrates (Vivelo et al., 2019). In one such example a complex comprising the RING E3 ligase DTX3L (Deltex-3-like) and the protein mono-ADP-ribosyltransferase PARP9 (Poly (ADP-ribose) polymerase 9) was shown to catalyze the attachment of ADP-ribose to the C-terminal glycine of ubiquitin (Figure 8C) (Yang et al., 2017). Initially assumed to be catalyzed by the ADP-ribosylation activity of PARP9, it was subsequently demonstrated that the conserved ∼230-residue RING-DTC (Deltex C-terminal) domains from DTX3L and other human Deltex family members (DTX1-4) were sufficient to catalyze the reaction themselves (Chatrin et al., 2020). Structural studies revealed that ubiquitin-loaded E2 (UBE2D2) bound to the ligase RING domain was brought into close proximity with NAD^+^ (nicotinamide adenine dinucleotide) bound to the DTC domain, facilitating the attachment of ADP-ribose to ubiquitin’s C-terminus. Therefore the exact mechanistic role of PARP9 in the DTX3L/PARP9 complex is unclear, but other Deltex ligases have also been reported to bind PARP enzymes (Ahmed et al., 2020; Wang et al., 2021), and heteromerization of DTX3L with PARP9 enhances both its canonical E3 ligase activity and its ADP-ribosyl transferase-like activity (Yang et al., 2017; Ashok et al., 2022). In addition to binding NAD^+^, the DTC domain of Deltex ligases can also bind to ADP-ribose, and it has been shown that the conventional ubiquitylation of target proteins by the Deltex family member DTX2 first requires their PARP1/2-mediated ADP-ribosylation (Ahmed et al., 2020), suggesting one possible role for PARP9 in the DTX3L/PARP9 complex.

The attachment of ADP-ribose to the C-terminus of ubiquitin has been observed in cells (Chatrin et al., 2020) but, as yet, no functions have been directly linked to this modification. It has been speculated however that conjugation of ADP-ribose to ubiquitin prevents conjugation of the ubiquitin to proteins by the canonical pathway (Chatrin et al., 2020). To date descriptions of the conjugation of ADP-ribose to ubiquitin have portrayed the reaction as an atypical form of ADP-ribosylation, but as the reaction can take place in the absence of recognized ADP-ribosyl transferases and strictly requires a three-enzyme cascade of E1 activating enzyme, E2 conjugating enzyme, and E3 ubiquitin ligase to attach the C-terminus of ubiquitin to an ADP-ribose substrate it may justifiably be considered as an example of non-canonical ubiquitylation. The reaction mechanism of such an unusual form of ubiquitylation would likely involve the prior hydrolysis of NAD^+^ into nicotinamide and ADP-ribose, possibly by the DTC domain, allowing the nucleophilic attack of the thioester bond by the 1′-hydroxy group of the now no longer nicotinamide-bound ribose moiety. Alternatively, the reaction may proceed like a canonical “substrate-assisted” ADP-ribosylation reaction with the C-terminal carboxylate group of ubiquitin acting as a nucleophile to attack NAD^+^. This mechanism would require hydrolysis of the E2-ubiquitin thioester bond in order to free ubiquitin’s C-terminus and permit nucleophilic attack, for which there is some evidence (Chatrin et al., 2020). Further mechanistic studies will be required to firmly establish which of these methods are used by Deltex family E3s to catalyze this remarkable form of ubiquitylation.

Ubiquitylated ADP-ribose can be recycled in cell lysates (Yang et al., 2017; Chatrin et al., 2020) and several linkage non-specific DUBs (Chatrin et al., 2020) and ADP-ribosylhydrolysases (Ashok et al., 2022) can remove ADP-ribose from ubiquitin *in vitro*. ADP-ribosylation is a phylogenetically ancient type of target-modifying signal that pre-dates the emergence of ubiquitylation and is conserved across all domains of life (Perina et al., 2014). The dynamic interplay between ubiquitin and ADP-ribose hinted at by these studies fits into a broader picture linking ADP-ribosylation and ubiquitylation, exemplified by the poly-ADP-ribosylation-dependent ubiquitylation of Axin by RNF146 (DaRosa et al., 2015) or the more recent findings that bacteria can catalyze the conjugation of ubiquitin to a serine or tyrosine residue in substrates via a phosphoribosyl linker—a novel type of non-lysine ubiquitylation known as phosphoribosyl (PR)-ubiquitylation (see below).

## Phosphoribosyl ubiquitylation in *Legionella pneumophilia*

**FIGURE 9:**
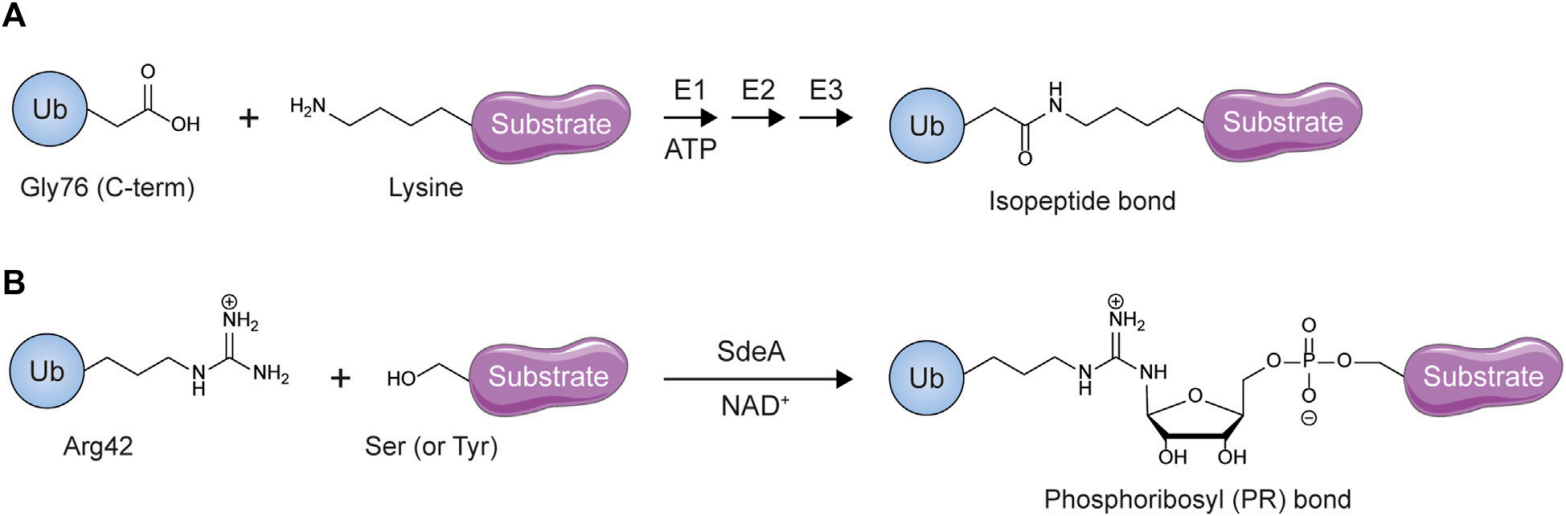
Unconventional ubiquitylation by Legionella pneumophilia. **(A)** Conventional eukaryotic ubiquitylation modifies substrate lysines. **(B)** PR-ubiquitylation is catalyzed by SidE effectors such as SdeA and targets substrate serines and tyrosines.

Bacteria lack a ubiquitin system of their own (Maupin-Furlow 2014). Nevertheless, they have evolved various highly effective strategies to hijack and co-opt the eukaryotic ubiquitin signaling system for their own needs (Berglund et al., 2020). In some instances this has led to the evolution of novel mechanisms that are significantly different from those observed elsewhere in nature. Members of the SidE effector protein family (SdeA, SdeB, SdeC and SidE) produced by *Legionella pneumophilia* bypass the classical three-enzyme cascade to catalyze a type of serine- and tyrosine-directed E1/E2-independent ubiquitylation known as phosphoribosyl (PR)-ubiquitylation (Figure 9) (Bhogaraju et al., 2016; Qiu et al., 2016; Kotewicz et al., 2017; Zhang et al., 2021). A combination of ADP-ribosyltransferase (ART) and phosphodiesterase (PDE) domains allows SidE-type enzymes to catalyze the conjugation of ubiquitin to substrate hydroxyl groups via a phosphoribosyl linker (Akturk et al., 2018; Dong et al., 2018; Kalayil et al., 2018; Kim et al., 2018; Wang et al., 2018). In the first step of a multi-step reaction the ART domain uses the nucleotide cofactor NAD^+^ to ADP-ribosylate ubiquitin on the side chain of Arginine 42. This intermediate is then recognized by the PDE domain, which uses a catalytic histidine nucleophile to cleave the high-energy phosphoanhydride bond between the two phosphate groups in ADP-ribosylated ubiquitin, generating Arg42 phosphoribosylated ubiquitin. This PR-ubiquitin is either transferred to serine or tyrosine residues in substrates or released from the enzyme’s active site due to hydrolysis. Interestingly, unconjugated PR-ubiquitin, much like ubiquitin conjugated to ADP-ribose via its C-terminus by Deltex ligases, cannot participate in conventional E1-mediated ubiquitylation and so poisons the host cell’s ubiquitylation machinery (Bhogaraju et al., 2016).

PR-ubiquitylation targets a large cohort of structurally diverse host proteins to promote bacterial proliferation, including proteins associated with ER remodeling, mitochondrial dynamics, autophagy, Golgi morphology, and the secretory pathway (Qiu et al., 2016; Kotewicz et al., 2017; Wan et al., 2019; Shin et al., 2020; Kawabata et al., 2021; Liu et al., 2021; Zhang et al., 2021). Structural analyses combined with the use of model target peptides suggests a promiscuous site selectivity in which disordered polypeptides with hydrophobic residues surrounding the target serine/tyrosine residue represent the preferred substrates (Akturk et al., 2018; Dong et al., 2018; Kalayil et al., 2018; Wang et al., 2018; Zhang et al., 2021). Unlike the other forms of non-canonical ubiquitylation discussed thus far, PR-ubiquitylation cannot be cleaved by conventional DUBs (Puvar et al., 2017). Instead, *Legionella* encodes two paralogous effectors called DupA and DupB (deubiquitylase for PR-ubiquitylation) that can specifically remove PR-ubiquitin from substrates (Wan et al., 2019; Shin et al., 2020). These effectors resemble the PDE domain of SdeA but display a higher affinity towards PR-ubiquitylated substrates, thereby mediating their DUP activity. Thus, *Legionella* can tune the level of PR-ubiquitylation in cells. Although some understanding has been gained, further studies will be required to fully appreciate how this extremely unusual form of non-lysine ubiquitylation is employed by *Legionella* to subvert the lost immune response and promote bacterial survival.

## Versatility and variety - New ways to regulate substrate fate

Lysine is one of the most modified amino acids (Azevedo and Saiardi 2016). So what advantage does nature derive from also targeting amino acid side chains containing hydroxy or thiol groups? Intuitively, this expansion of the ubiquitin code extends the biological possibilities open to the ubiquitylation machinery as well as offering up new methods for regulation and crosstalk with the canonical lysine-directed ubiquitin system. Several examples of this have been detailed during the course this review. For example, cysteine ubiquitylation of PEX5 following peroxisomal cargo import can act as a redox-sensitive switch to allow cells to rapidly respond to oxidative stress. The same ubiquitylation machinery that ubiquitylates PEX5 on Cys11 can also degrade PEX5 by ubiquitylating it by conventional means on lysine residues (reviewed, Wang and Subramani 2017), highlighting not only the interplay that can exist between canonical and non-canonical ubiquitylation but also the promiscuity that seems to be a feature of the ubiquitylation machinery involved in non-canonical ubiquitylation. UBE2W, UBE2J2, RNF213, and the Deltex ligases have all been reported to catalyze lysine-directed ubiquitylation in addition to their non-canonical activities. Such catalytic plasticity is not uncommon amongst enzymes (Khersonsky and Tawfik 2010) and is consistent with mutagenesis data from both ERAD and PEX5 recycling that suggests that it is the *position* of the ubiquitylated residue and not its identity that frequently determines whether ubiquitylation occurs or not. Given the structural diversity and potential quantity of substrates that UBE2J2 may have to ubiquitylate during ERAD (potentially upwards of 7,000 unique proteins) this target residue flexibility may be advantageous in permitting a more opportunistic mode of action that allows arbitrary substrate proteins to be degraded in a timely manner even if available lysine residues are sparse - a sort of molecular failsafe mechanism. That said, the relationship between ERAD and infectious pathogens can often resemble a molecular arms race (Byun et al., 2014; Morito and Nagata 2015; Frabutt and Zheng 2016) and several AB-toxins, a class of ERAD-subverting proteins produced by both plants and bacteria, possess a remarkable arginine-over-lysine bias suggested to help them escape ubiquitin-mediated degradation during retrotranslocation to the cytosol (London and Luongo 1989; Hazes and Read 1997; Deeks et al., 2002; Rodighiero et al., 2002; Worthington and Carbonetti 2007). The PST1 subunit of the pertussis toxin, for example, is a naturally lysine-less protein (Locht and Keith 1986; Nicosia et al., 1986). Replacing up to three of PST1’s twenty-two arginines with lysines has no effect on *in vitro* enzymatic activity but reduces PST1’s *in vivo* activity by promoting increased proteasomal degradation (Worthington and Carbonetti 2007). A similar enhancement of proteasomal degradation is observed upon introduction of four additional lysines into the plant toxin Ricin, but interestingly mutation of its two endogenous lysines has no discernable effect on degradation, suggesting that the protein’s endogenous lysine residues are not the usual sites of ubiquitylation (Deeks et al., 2002). Whether or not ERAD substrates are ubiquitylated by priming upon canonical or non-canonical residues may depend not only upon the residues available in the substrate, but also the E3 tasked with ubiquitylating them. The viral ligase mK3 shows a preference for serine and threonine residues in the MHC-I heavy chain even when lysines are available (Wang et al., 2007; Wang et al., 2009), whereas the ERAD ligase HRD1 can ubiquitylate MHC-I HC on both lysine and non-lysine residues but shows a preference for lysine (Burr et al., 2013). Lysine and non-lysine ubiquitylation can, in some cases, lead to different kinetics of target protein degradation during ERAD (Ishikura et al., 2010; Burr et al., 2013) suggesting differential handling of these modifications by the ubiquitin-proteasome machinery. Similarly, alterations in proteasomal degradation are also observed when Cys11 in PEX5 is mutated to lysine, encouraging polyubiquitylation of the peroxisomal cargo receptor rather than monoubiquitylation (Schwartzkopff et al., 2015).

## Interplay between ubiquitylation and phosphorylation

Perhaps one of the most obvious benefits of expanding ubiquitylation to include residues beyond lysine is that it affords the opportunity for crosstalk with additional post-translational modifications (PTMs), at the same time avoiding competition with lysine-targeted PTMs such as acetylation, which has been reported to prevent substrate ubiquitylation and suppress isopeptide-linked ubiquitin chain formation (Gronroos et al., 2002; Ito et al., 2002; Vervoorts et al., 2003; Bernassola et al., 2004; Jin et al., 2004; Simonsson et al., 2005; Le Cam et al., 2006; Min et al., 2010; Ohtake et al., 2015; Fan et al., 2022). The complex interplay between ubiquitylation and protein phosphorylation is well established and has been extensively reviewed elsewhere (see, for example, Hunter 2007; Chen and Chen 2013; Nguyen et al., 2013; Cohen 2014; Schwertman et al., 2016; Filipcik et al., 2017; Song and Luo 2019; Zhang and Zeng 2020; Dang et al., 2021; Lacoursiere et al., 2022). Therefore, the ability of ubiquitin to target the same hydroxylated residues as cellular kinases is an exciting development. In much the same way that acetylation can inhibit substrate ubiquitylation by competing for modification of the same lysine residue, it is possible to envisage a situation where prior phosphorylation of a serine or threonine could prevent its oxyester-linked ubiquitylation (or vice-versa). HOIL-1 was identified in rat brain by means of a yeast two-hybrid screen using Protein Kinase C (PKC) β as bait (Tokunaga et al., 1998). It was subsequently shown that phosphorylation of HOIL-1 by PKCβ *in vitro* prevented its autoubiquitylation (Tatematsu et al., 2008). Perhaps tellingly, one of the *in vitro* phosphorylation sites identified in HOIL-1, Ser137, has also been reported as a site of non-canonical HOIL-1 autoubiquitylation (Kelsall et al., 2019), hinting that competition at this site may result in mutually exclusive modifications. The ability of HOIL-1 to form oxyester-linked ubiquitin dimers and introduce branch points in polyubiquitin chains also offers the tantalizing prospect of ubiquitin chain formation regulated by competing phosphorylation on serines and threonines within ubiquitin itself. Proteomic studies have detected phosphorylation of most of the serine, threonine, and tyrosine resides in ubiquitin (summarised in Herhaus and Dikic 2015; Swatek and Komander 2016; Hepowit et al., 2021) (Figure 7), although the functional relevance, site stoichiometries, and kinases and phosphatases responsible for these phosphorylations are largely unknown. By far the best studied example of ubiquitin phosphorylation is the phosphorylation of Ser65 catalyzed by PINK1 (PTEN induced kinase 1) during mitochondrial autophagy. Allosteric binding of this Ser65-phosphorylated ubiquitin to the E3 ubiquitin ligase Parkin is required to relieve autoinhibition and achieve maximal Parkin activation during oxidative stress models of mitophagy (reviewed, Bayne and Trempe 2019; Tang and Zhang 2020). Recently it has been reported that ubiquitin is phosphorylated at Thr12 during the DNA damage response (Walser et al., 2020). This is relevant because HOIL-1 produces ubiquitin dimers conjugated at the Thr12 position (Kelsall et al., 2019; Rodriguez Carvajal et al., 2021). Threonine-12 is one of just sixteen ubiquitin residues that are essential for life in *S. cerevisiae* (Sloper-Mould et al., 2001) and forms part of a ubiquitin surface feature known as the TEK box (Jin et al., 2008; Kulathu and Komander 2012), highlighting its importance in ubiquitin biology. It will be interesting to see if phospho-Thr12 occurs in response to other cellular stimuli and to establish if there is indeed antagonism with ubiquitylation at the same site.

## Is non-proteinaceous ubiquitylation a widespread phenomenon

A further PTM that might compete with non-lysine ubiquitylation at serine and threonine residues is *O*-GlcNAcylation, a type of non-canonical nucleocytoplasmic glycosylation that involves the attachment of a single *N*-acetylglucosamine sugar moiety to Ser/Thr residues in target proteins via a β-*O*-glycosidic linkage (Yang and Qian 2017). As it targets serine and threonine, there is a well-established crosstalk between *O*-GlcNAcylation and phosphorylation (Hart et al., 2011), but studies have also revealed an extensive crosstalk between *O*-GlcNAcylation and protein ubiquitylation (reviewed in Ruan et al., 2013). In addition to the ability of non-lysine ubiquitylation to compete with *O*-GlcNAcylation for modification of serine and threonine residues, the recent finding that HOIL-1 can ubiquitylate glucosaccharides gives rise to the intriguing idea that *O*-GlcNAcylation might in fact create a new ubiquitylation site that can be targeted by a specific sugar-directed ubiquitin ligase. Protein glycosylation is typically considered to happen outside of the nucleocytoplasmic milieu, but in reality glycoproteins can, and do, appear in the cytosol, where they serve as a trigger to promote ubiquitylation and degradation of unwanted proteins and organelles (Yoshida and Tanaka 2018). The ubiquitylation of LPS by RNF213 is an example of the fate that may await a bacterial glycan in the cytosol, but in addition to exogenous bacterial and viral surface-exposed glycans, endogenous glycoproteins can appear in the cytosol as a result of organelle damage or as a consequence of retrograde transport of glycoproteins from the ER to the cytosol during ERAD. It is tempting to speculate that the emergence of high-mannose glycans into the cytosol in a process involving the highly promiscuous non-canonical ubiquitin conjugating enzyme UBE2J2 might create a situation where further non-protein ubiquitylation can occur. In addition to enzymatic glycosylation, proteins may also be glycated non-enzymatically by reducing sugars such as glucose to form lysine-bound adducts known as Schiff bases or Amadori products which, if left unchecked, can proceed to irreversibly form dangerous advanced glycation end products (Popova et al., 2010). Organisms contain natural defenses against the formation and accumulation of advanced glycation end products, but once more it is tempting to speculate that ligases might exist that can recognize and degrade proteins containing the Schiff base or Amadori intermediates by directly ubiquitylating the sugar moiety to prevent progression to advanced glycation end products. Undoubtably, the field has many surprises to reveal in the coming years as we learn more about non-proteinaceous ubiquitylation, but such discoveries will require researchers to transition into areas not normally associated with ubiquitylation research and embrace technologies such as metabolomics, glycomics, and lipidomics. Thus far evidence of non-protein ubiquitylation has come about fortuitously during the course of studying a specific protein or system of interest, but targeted global screens will be required to truly gauge how widespread this phenomenon is. The development of linkage-specific antibodies and tailored methods for enrichment will also be necessary to facilitate discovery in the same way that they have for conventional lysine-directed ubiquitylation.

## Experimental approaches to probe non-canonical ubiquitylation - The pitfalls and the prospects

The labile nature of thioester and oxyester bonds means that current experimental methods may easily miss evidence of non-canonical ubiquitylation. Something as simple as running SDS-PAGE gels under reducing and non-reducing conditions reveals how easily cystine-targeted ubiquitylation might be missed under normal gel running conditions (Tait et al., 2007; Vosper et al., 2009). And whereas the susceptibility of the oxyester bond to hydrolysis under mild alkaline conditions proves a simple way to test whether ubiquitylation is isopeptide- or oxyester-linked (Wang et al., 2007; Pao et al., 2018; Kelsall et al., 2019), this pH-dependent lability can also make this modification hard to detect by methods such as mass spectrometry or gel analysis if the sample is not carefully prepared and consideration taken to avoid heating and high pH (Shimizu et al., 2010; Wang X. et al., 2012; Anania et al., 2013; Lei et al., 2018).

Mass spectrometry is usually considered the gold standard to identify protein ubiquitylation sites (Steger et al., 2022) but unless machines are specifically programmed to search for non-lysine ubiquitylation this information is simply lost amongst the thousands of unassigned spectra (Pathan et al., 2017). Ubiquitin proteomics is typically accompanied by an enrichment step, often involving purification using a linkage-specific ubiquitin binder (Hjerpe et al., 2009; Emmerich et al., 2013; Michel et al., 2017; Antico et al., 2021) or peptide enrichment using an antibody recognizing the diGly remnant attached to ubiquitylated lysines following tryptic digest (Xu et al., 2010). As discussed earlier regarding N-terminal ubiquitylation, this antibody is unlikely to enrich peptides modified on residues other than lysine. To date no ubiquitin-binding domain has been identified that specifically recognizes non-lysine ubiquitin linkages but, based upon the close association between HOIL-1-catalysed oxyester-linked ubiquitin and HOIP-catalyzed Met1-linked ubiquitin chains, the Met1-linked ubiquitin binding protein NEMO has been used to purify and detect HOIL-1 substrates for western blotting (Kelsall et al., 2019; Petrova et al., 2021). This approach could perhaps be adapted to enrich oxyester ubiquitylated proteins prior to mass spectrometry, but would likely need to be paired with a peptide enrichment step akin to the anti-K-ε-GG antibody. Blagoev and colleagues have developed a strategy known as UbiSite that uses an antibody recognizing a 13 amino acid remnant generated by LysC digestion of ubiquitylated proteins (Akimov et al., 2018a). Unlike the anti-K-ε-GG antibodies, UbiSite should recognize proteins (and other biomolecules) ubiquitylated on residues other than lysine and has already improved the detection of N-terminal ubiquitylated substrates when compared to conventional methods (Akimov et al., 2018a).

An important step in understanding the role and extent of non-lysine polyubiquitylation in cells will be identifying the ‘readers’ - the ubiquitin binding proteins and domains that detect oxyester linkages within polyubiquitin chains. This may be achieved by synthesizing oxyester-linked ubiquitin chains either enzymatically or chemically and identifying binding partners *in vitro* and in cell lines (Komander et al., 2009; Kristariyanto et al., 2015; Zhang et al., 2017; Zhao et al., 2017). To date there is one example of chemically synthesized threonine-linked ubiquitylation (Sun et al., 2018) and it will be important to develop further chemical-biological tools to probe non-canonical ubiquitylation, such as non-hydrolysable oxyester-linked chains and chemically modified substrates non-canonically ubiquitylated at a defined site (Gui et al., 2021). Such methods will also be instrumental in delineating the role of non-protein ubiquitylation through the creation of chemically ubiquitylated sugars and lipids (see Squair and Virdee 2022 for further discussion of how chemical biology could be used to better understand non-lysine ubiquitylation).

## Concluding remarks

During the mid-1970s a remarkable bifurcated histone adduct was discovered in which the C-terminus of the small heat-stable protein ubiquitin was linked via an isopeptide bond to the ε-amino group of an internal lysine in the histone protein (Goldknopf and Busch 1977). Subsequent experiments over the following two decades reinforced the notion that ubiquitylation always involved the covalent attachment of ubiquitin only to lysine residues in substrate proteins (Pickart 2001). But, since the late 1990s onwards the field has seen an accumulation of evidence in support of, first N-terminal, and then ester-linked, ubiquitylation. More recently examples have emerged that extend the reach of ubiquitylation to non-proteinaceous substrates such as sugars and lipids. Indeed, even while the revised version of this manuscript was in preparation a new study emerged from the Mizushima lab reporting the conjugation of ubiquitin to phospholipids in yeast and mammalian cells (Sakamaki et al., 2022), suggesting that RNF213 is not the only ligase with lipid-directed activity. Results from bacteria have also completely rewritten the rules of ubiquitylation, utilizing novel reaction mechanisms and linkage types to couple ubiquitin to substrates. Of the more than 330 effector proteins that *Legionella pneumophilia* secretes into the host cytosol, the functions and biochemical activities of most remain a mystery (Schroeder 2017), meaning that there is still significant opportunity for further surprises. Does *Legionella* also target non-protein substrates such as membrane lipids or cellular metabolites to promote its survival? And are there human equivalents to SidE that can PR-ubiquitylate substrates in the absence of recognized components of the canonical ubiquitylation cascade? The answers to these, and other questions, will become apparent in the coming years, no doubt driven by the development of improved technologies and an enhanced willingness by researchers to look beyond lysine when considering E3 ligase targets.

The realization that so many mammalian DUBs display esterase activity, and that some even seem to preferentially target oxyester linkages over isopeptide bonds, indicates that non-lysine ubiquitylation is likely to represent a general feature of mammalian ubiquitylation. UBE2J2 and UBE2W represent the two E2s with confirmed non-lysine activity and both lack key residues normally associated with aminolysis of the substrate lysine. Interestingly though, they are not the only E2s to lack these conserved features of canonical ubiquitylation. UBE2Q1 and UBE2Q2 stand out as two poorly characterized E2s that lack both the HPN motif and the conserved downstream D/S residue (Figure 4). Like UBE2J2, these enzymes are active even in the absence of a specific E3 ligase (De Cesare et al., 2018). Could they represent further examples of non-lysine targeted E2s?

And what of the E3s? It is notable that MYCBP2 utilizes a ubiquitin relay mechanism for catalysis while a similar coordinated relay mechanism has also been proposed to operate in LUBAC. It is possible that such a feature may be a hallmark of those E3s that catalyze ester-linked ubiquitylation. Only as we uncover more examples of ligases directed against non-lysine residues will we be able to know for sure.

Charles Darwin concluded *‘On the Origin of Species’* by marveling at how nature could create *‘endless forms most beautiful and most wonderful’* from the most simple of mechanisms and building blocks (Darwin 1859). With these words Darwin could have been describing ubiquitin. This deceptively simple protein continues to amaze with its versatility and with the myriad ways in which it can be conjugated to itself and its substrates. The last few years have seen important advances in our appreciation and understanding of non-canonical ubiquitylation. It is highly likely that the coming years will see further advances that will continue to re-write the rules and challenge the existing paradigms within the field. As we move ever closer to the 50th anniversary of ubiquitin’s discovery in 1975, this remarkable protein continues to astound!

## Known unknowns—some outstanding questions

1) How prevalent is non-lysine ubiquitylation?
2) Do ester-linked ubiquitin chains exist within cells?
3) Is the ubiquitylation of non-proteinaceous substrates (lipids, sugars) a general mechanism for the detection and clearance of invading pathogens such as bacteria and viruses?
4) Is glycogen ubiquitylated *in vivo* and what physiological role does this play?
5) What structural and biochemical features determine the reactivity of E2s, E3s, and DUBs towards non-lysine residues and non-proteinaceous substrates?
